# Spontaneous Strain‐Spin Transition Coupling Molecular Crystal with Thermal Magnetic Memory Effect, Anisotropic High‐κ and Switchable Dielectric Permittivity

**DOI:** 10.1002/advs.202501925

**Published:** 2025-06-04

**Authors:** Xuan‐Rong Chen, Zhang‐Ni He, Yin Qian, Wei Wei, Zheng‐Fang Tian, Xiao‐Ming Ren

**Affiliations:** ^1^ State Key Laboratory of Materials‐Oriented Chemical Engineering and College of Chemistry and Molecular Engineering Nanjing Tech University Nanjing 211816 P. R. China; ^2^ School of Chemistry & Environmental Engineering and Instrumental Analysis Center Yancheng Teachers University Yancheng 224007 P. R. China; ^3^ Hubei Key Laboratory for Processing and Application of Catalytic Materials Huanggang Normal University Huanggang 438000 P. R. China; ^4^ State Key Laboratory & Coordination Chemistry Institute Nanjing University Nanjing 210023 P. R. China

**Keywords:** anisotropic dielectric permittivities, high‐κ, magnetic thermal memory effects, mirror symmetry breaking, paraelastic‐ferroelastic phase transitions

## Abstract

Materials that exhibit controllable changes in electrical, magnetic, or spontaneous strain properties, particularly those that couple these functionalities simultaneously, hold significant potential for technological applications. In this study, a 1D phase transition ion‐pair compound is investigated, triethylmethylammonium bis(1,2‐maleonitriledithiolato)nickelate (abbr. [Et_3_MeN][Ni(mnt)_2_], **1**), composed of flexible Et_3_MeN^+^ cation and planar radical [Ni(mnt)_2_]^−^ anion. This salt undergoes a paraelastic‐ferroelastic phase transition at ≈233/224 K (on heating/cooling), driven by spin‐lattice interactions. Importantly, the phase transition couples spontaneous strain, bistable magnetism with switchable dielectric properties. Another distinctive feature of **1** is its pronounced dielectric anisotropy and high dielectric permittivity, which arise due to a barrier layer capacitor effect due to cation displacement polarization and significant electron polarization of the highly conjugated anions. These findings provide a versatile molecular design strategy for developing magnetoelectric and mechanically multifunctional materials, with promising applications in next‐generation electronic and smart devices that leverage coupled physical properties.

## Graphical abstract

A radical salt undergoes a paraelastic‐ferroelastic phase transition, which is coupled with spin‐Peierls transition, resulting in it integrating self‐strain, spin bistability, switchable dielectric and high‐ properties.

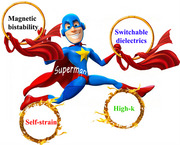

## 1 Introduction

The development of advanced materials with engineered spontaneous strain, different magnetic, and dielectric properties is essential for the next generation of electronic, spintronic, and memory devices,^[^ ^1^, ^2^, ^3^ ^]^ as well as for capacitors, sensors, and actuators.^[^ ^4^, ^5^, ^6^ ^]^ Among these, materials with switchable magnetic or dielectric properties have garnered significant research interest, because these materials can reversibly modify their magnetic susceptibility or dielectric constants in response to external stimuli, such as electric fields, temperature variations, or mechanical stress. The ability to switch between different magnetic or dielectric properties offers considerable potential for memory applications.^[^ ^3^, ^7^ ^]^ Notably, materials that exhibit both switchable magnetic and dielectric properties provide dynamic tunability and can retain distinct magnetic and electric states simultaneously, thereby facilitating multilevel information storage.^[^ ^8^ ^]^

The molecule‐based switchable magnetic materials include spin‐crossover (SCO),^[^ ^9^, ^10^, ^11^, ^12^ ^]^ valence tautomeric,^[^ ^13^, ^14^ ^]^ and radical compounds containing spin dimers^[^ ^8^ ^]^ or spin chains.^[^ ^15^, ^16^ ^]^ Switchable dielectric behaviors in molecule‐based materials are often associated with structure transformations.^[^ ^10^, ^17^ ^]^ Despite the distinct mechanisms of switchable magnetic or dielectric properties, these materials share a common characteristic: the switchable behavior is typically coupled with a phase transition. The coexistence of dielectric and magnetic bistability is particularly valuable for applications that utilize coupling between magnetic and dielectric states in multifunctional device architectures. However, such multifunctional materials have been rarely reported to date.^[^ ^3^, ^10^, ^18^, ^19^, ^20^, ^21^ ^]^

Anisotropic dielectric permittivity and high dielectric permittivity (high‐κ) are two critical functionalities in materials science. The former, characterized by a directional dependence of the dielectric response, enables selective control over dielectric properties, thereby enhancing efficiency and energy storage capabilities. This is achieved through the optimization of permittivity along specific crystallographic axes, making it particularly useful in applications such as capacitors, sensors, and actuators. Typical examples of anisotropic dielectric materials include highly anisotropic crystalline systems.^[^ ^22^, ^23^, ^24^, ^25^ ^]^

High‐κ materials, on the other hand, play a crucial role in modern electronics and energy storage applications.^[^ ^26^, ^27^ ^]^ Numerous high‐κ materials have been identified, with their properties often linked to intrinsic or relaxor ferroelectricity.^[^ ^28^ ^]^ High‐κ behavior has also been observed in non‐ferroelectric systems, where these properties arise through alternative mechanisms, including extrinsic effects such as internal barrier layer capacitance (IBLC), commonly observed in CaCu_3_Ti_4_O_12_ (CCTO),^[^ ^29^ ^]^ barrier layer capacitors associated with grain boundary effects in ion‐conducting materials,^[^ ^30^, ^31^ ^]^ and charge‐density waves (CDW) in low‐dimensional materials. In CDW systems, the charge density forms a periodic modulation, which may be incommensurate with the crystal lattice, as observed in certain metal‐insulator transition compounds. ^[^ ^32^, ^33^ ^]^

Herein, we investigated the radical salt, [Et_3_MeN][Ni(mnt)_2_] 1). The [Ni(mnt)_2_]^−^ anions tend to form stacked structures, exhibiting an S = ½ magnetic chain configuration in the lattice, which often undergo spin‐Peierls‐type transitions driven by spin‐lattice interactions. In contrast, the globular‐shaped Et_3_MeN^+^ cations demonstrate volume‐conserving motion,^[^ ^17^, ^34^ ^]^ with lower rotational energy barriers in the lattice,^[^ ^35^ ^]^ enabling rotational dynamics through thermal activation and resulting in disorder‐order phase transitions.^[^ ^35^, ^36^ ^]^ As expected, the [Ni(mnt)_2_]^−^ anions stack into a 1D spin chain in the crystal. Furthermore, this molecular crystal undergoes a paraelastic‐to‐ferroelastic phase transition, characterized by mirror‐symmetry breaking, coupled with magnetic memory effects and switchable dielectric behavior, exhibiting anisotropic high‐κ properties.

## 2 Results and Discussion

### 2.1 Crystal Structures, DSC and Ferroelastic Property

Compound **1** undergoes a phase transition at 224/233 K (upon cooling/heating). Accordingly, its crystal structures were determined at 293, 270, and 250 K in the high‐temperature phase (HTP), and at 150, 125, and 100 K in the low‐temperature phase (LTP). In this study, we provide a detailed description of the crystal structures at 293 K in the HTP and at 100 K in the LTP. The crystal structure information at other temperatures is summarized in Table S1 (Supporting Information).

**Figure 1:**
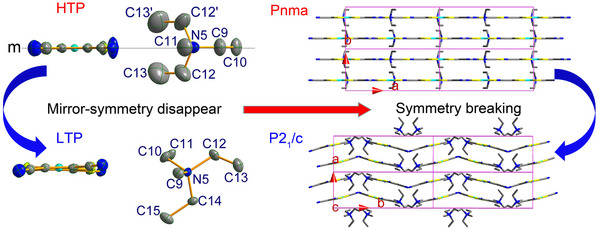
ORTEP view drawn at the 20% probability level at 293 K in the HTP and the 50% probability level at 100 K in the LTP, as well as illustration for molecular mirror symmetry disappearance and crystal packing structure change from HTP to LTP for **1**.

In the HTP, the crystal of **1** belongs to the orthorhombic space group *P*nma, with the asymmetric unit consisting of a pair of [Ni(mnt)_2_]^−^ anions and Et_3_MeN^+^ cations (**Figure** **1**; Figures S1 and S2, Supporting Information). The bond lengths and angles (Table S2, Supporting Information) are comparable to those in reported compounds containing the [Ni(mnt)_2_]^−^ anion^[^ ^35^ ^]^ and the Et_3_MeN^+^ cation.^[^ ^37^ ^]^ The N5, C9, C10, C11 atoms in the cation moiety and all atoms in the anion moiety are located on a mirror plane with Wykoff site 4c, resulting in both the anion and cation with the symmetry of the C_S_ point group. The anions form stacks running along the b‐axis, with the cations residing in the spaces of inter‐anion‐stacks (Figure 1; Figures S1 and S2, Supporting Information). Within a stack, two superimposed anions are shifted along one Ni–S bond direction, with identical Ni…Ni distances (4.116 Å). The neighboring anion stacks show the same orientation along the a‐axis, but alternating between two different orientations along the c‐axis (Figures S1 and S2, Supporting Information).

Upon transitioning from HTP to LTP, the phase transformation results in the crystal structure changing from the orthorhombic system to the monoclinic system (*P*2₁/c), and both the anion and cation losing their mirror symmetry, and the phase transition is characterized by mirror symmetry‐breaking. The asymmetric unit remains composed of a pair of anion and cation (Figure 1; Figures S1 and S2, Supporting Information), while all atoms locate at a general position. Compared to the HTP, the crystal structure changes in the LTP involve the following aspects: 1) Distortion occurs within the anion stack, leading to dimerization of the stack and a shift of neighboring anions along the long molecular axis (Figure 1; Figures S1 and S2, Supporting Information), giving two types of adjacent Ni…Ni distances (4.407/3.975 Å). 2) The neighboring anion stacks adopt a herringbone form in the LTP, whereas a block‐wall shape in the HTP along the long molecular axis (corresponding to the b‐axis direction in the LTP while the c‐axis direction in the HTP, Figure 1; Figures S1 and S2, Supporting Information). 3) The cation rotates ≈120° around the anion stack. The movements of both anions and cations in the lattice during the transition result in the single crystal of **1** transforming into a twinned crystal in the LTP.

**Figure 2:**
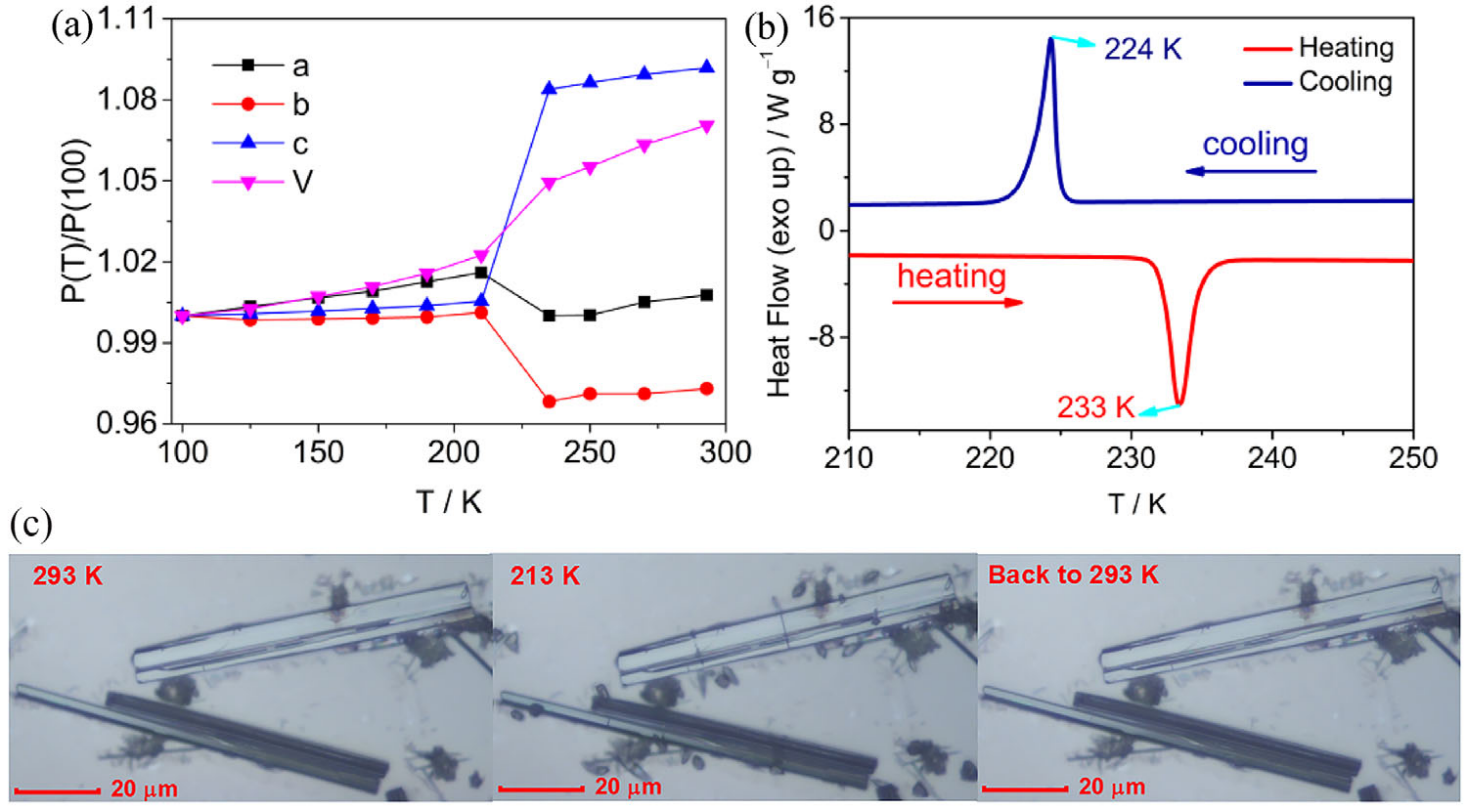
a) Relative cell parameters change with temperature, b) DSC curves, c) image of hot‐stage polarization microscopy showing ferroelastic domain evolution with temperature change for **1**.

The phase transition results in the a, b, and c axes of the crystal in the HTP transforming into the c, −a, and −b axes in the LTP, respectively. Temperature‐dependent cell relative parameters were recorded in 100–293 K for **1** are defined as P(T)/P(100), where P(T) and P(100) represent the cell parameters—the lengths of the a, b, and c axes, and the cell volume—at temperature T and at 100 K, respectively. The P(T)/P(100) as a function of temperature is plotted in **Figure** **2**a. All relative cell parameters exhibit a sharp change around T_C_ (235 K), with the first‐order phase transition characteristic.

From the LTP to the HTP, the crystal axes of **1** show anisotropic variation. The length corresponding to the a‐axis in the LTP and the b‐axis in the HTP contracts slightly. In contrast, the length corresponding to the b‐axis in the LTP and the c‐axis in the HTP shrinks markedly, with a 3.3% decrease from 210 to 235 K. Meanwhile, the length corresponding to the c‐axis in the LTP and the a‐axis in the HTP expands by 7.2% over the same temperature range. During this phase transition, the cell volume expands by 2.6% from 210 to 235 K. As shown in Figure 1, Figures S1 and S2 (Supporting Information), significant expansion and contraction occurred in the directions perpendicular to the anionic stacks due to relative displacements between neighboring anionic stacks during the phase transition. This structural change makes the crystal of **1** prone to breaking, which is the primary reason why the single crystal exhibits twin characteristics when cooled from the HTP to the LTP.

DSC plots of **1** reveal a pair of thermal anomaly peaks at 233 K during the heating and at 224 K during cooling, with an observed thermal hysteresis of ≈7 K (Figure 2b). The change in enthalpy (ΔH) of the phase transition is determined to be ≈5.6 kJ mol^−1^, and the change in entropy (ΔS), calculated using ΔS = ΔH/T_C_ (where T_C_ = 233 K), is 24.2 J K^−1^ mol^−1^ for the heating process. Both the thermal hysteresis and latent heat further confirm the first‐order phase transition characteristic, consistent with the discontinuous changes in cell parameters near the T_C_.

Since structural phase transition occurs between *P*nma and *P*2_1_/c, belonging to one of the 94 species of ferroelastic phase transitions, with an Aizu notation of mmmF2/m.^[^ ^38^ ^]^ A hot‐stage polarization microscopy was used to observe the domain structure evolution of crystals. On cooling, the domain patterns occur at 213 K (LTP) and the stripes clearly appear on the surface of the crystals, corresponding to different orientation states (Figure 2c). Upon heating, the stripes vanished. ^[^ ^39^ ^]^

The spontaneous strain components were calculated based on the lattice parameters. For an mmmF2/m system, two orientational states (S₁ and S₂) correspond to spontaneous strain tensors with shear components involving 𝑥_13_, which can be calculated using Equation (1):

(1) $$x_{13}=\frac{1}{2}\;\left(\frac{c\cdot cos\beta }{c_{0}\cdot sin\beta_{0}}-\frac{a\cdot cos\beta_{0}}{a_{0}\cdot sin\beta_{0}}\right)$$

In Equation (1), a, c, β and a_0_, c_0_, β_0_ are the cell parameters of **1** in the LTP (150 K) and HTP (250 K), respectively, and the matrix transformation relating the HTP and LTP axes is provided in the Supporting Information. The spontaneous strain x_s_ = $\sqrt{2}$ x_13_ is estimated as 0.0029 for **1**, less than that of other hybrid ferroelastics.^[^ ^40^, ^41^, ^42^, ^43^, ^44^, ^45^ ^]^

### 2.2 Magnetic Bistable Behaviour

**Figure 3:**
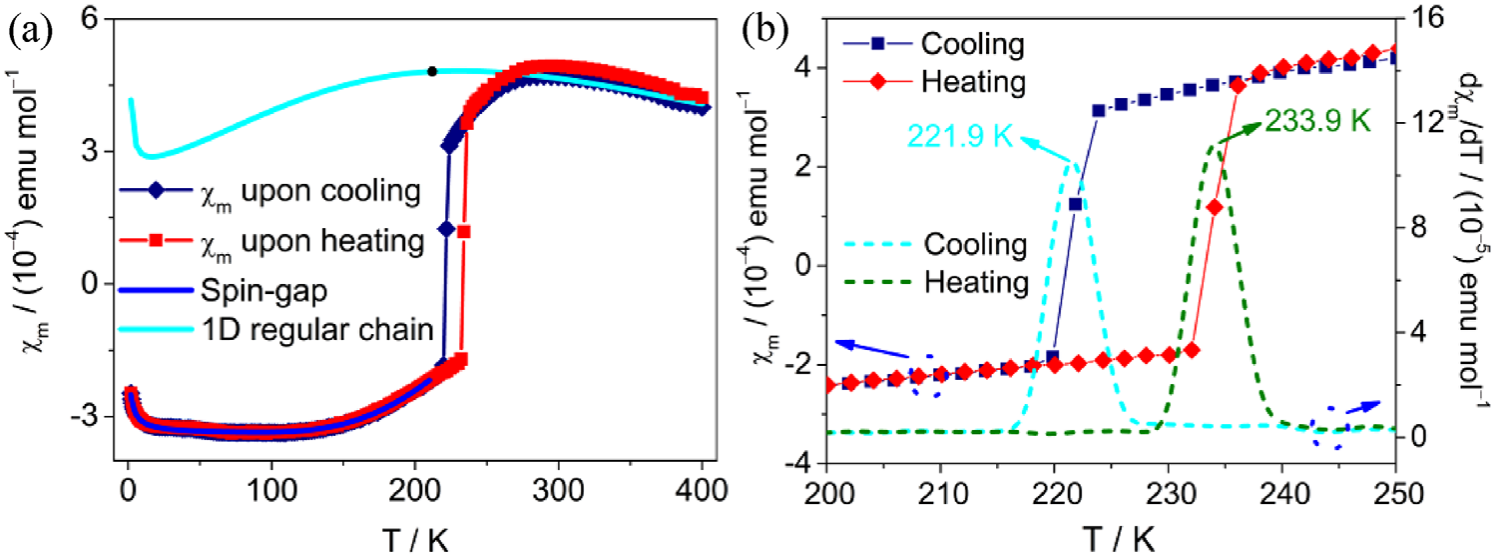
a) Temperature‐dependent molar magnetic susceptibility (χ_m_) of **1** during cooling and heating from 1.8 to 400 K. Fitting data through spin‐gap equation in the temperature range of 1.8−208 K in the LTP and 1D‐uniform spin chain in the HTP (270–400 K), b) the enlarged sections show the thermal hysteresis loop for **1**.

The *χ*_m_ versus *T* plots of **1** during both cooling and heating in 1.8–400 K are displayed in **Figure** **3**a, here, *χ*_m_ represents the molar magnetic susceptibility with one [Ni(mnt)_2_]^−^ anion per formula unit. A broad maximum, characterized by a low‐dimensional antiferromagnetic (AFM) system, appears ≈285 K, indicating the presence of short‐range AFM couplings in **1**. Upon further cooling, the magnetic susceptibility sharply drops ≈224 K, reaching a negative value and decreasing exponentially with temperature in 220–100 K, demonstrating that **1** underwent a magnetic transition and entered a spin gap phase in the LTP. Below ≈100 K, compound **1** exhibits Curie‐Weiss type magnetic behavior. Upo heating, a sudden jump in magnetic susceptibility is observed at 233.2 K. During both heating and cooling, the *χ*_m_ versus *T* plots are nearly superimposed, except near the temperature range of 225–233 K (Figure 3a,b). The d*χ*_m_ versus *T* plots are displayed in Figure 3b, where two peaks at T_↓_ = 221.9 K and T_↑_ = 233.9 K correspond to the sharp drop and jump in magnetic susceptibility in the *χ*_m_ versus *T* plots during cooling and heating. The hysteresis loop, estimated as ΔT = T_↑_ − T_↓_ = 6.4 K, is comparable to the results obtained from the DSC technique.

In the LTP, the magnetic susceptibility of **1** change with temperature shows spin gap feature, which was fitted by Equation (2):

(2) $$\chi_{m}=\frac{\alpha }{T^{\gamma }}\cdot \exp\left(-\frac{\Delta }{k_{B}T}\right)+\frac{C}{T-\theta }+\chi_{0}$$

Here, α represents a constant corresponding to the dispersion of excitation energy. For a 1D S = ½ linear chain system with a spin gap, *γ* = ½ and *Δ/k_B_* signifies the magnitude of the spin gap.^[^ ^46^ ^]^ *C* and *θ* denote the Curie and Weiss constants, respectively, while *χ_0_* accounts for contributions from core diamagnetism and potential van Vleck paramagnetism. The best fits for the magnetic susceptibility data of **1** in 1.8–208 K yielded the parameters: α = 0.2(2), Δ/k_B_ = 1016(4) K, C = 3.3(1) × 10^−3^ emu K mol^−1^, θ = −1.7(2), and *χ_0_* = −3.4(1) × 10^−4^ emu mol^−1^ (Figure S3, Supporting Information). The larger Δ/k_B_ values are consistent with the observation that the diamagnetism dominates the magnetic behavior of **1** in the LTP. The smaller Curie and Weiss constants indicate weak Curie‐Weiss type magnetic behavior at the temperatures below 100 K, caused by uncoupled spins at lattice defects. The χ_0_​ term is comparable to the diamagnetic susceptibility estimated from Pascal's constants, which generally exhibit values of about −M×10^−6^ emu mol^−1^ (where M represents the molecular weight).

An S = ½ AFM Heisenberg regular linear chain model is employed for the magnetic susceptibility analysis of **1** in the HTP, owing to the regularity of the anion stack. For the Heisenberg regular linear chain, the spin Hamiltonian is expressed as shown in Equation (3):

(3) $$\hat{H}=-2J\sum_{i=1}^{n}{\hat{S}}_{i}{\hat{S}}_{i+1}$$

where J represents the magnetic exchange constant between neighboring spins, with J < 0 indicating an AFM exchange system.^[^ ^47^, ^48^, ^49^ ^]^ The temperature‐dependent *χ*_m_ for an S = ½ Heisenberg AFM regular linear chain, derived using the cluster approach, is given by:

(4) $$\chi_{chain}=\frac{Ng^{2}\mu_{B}^{2}}{k_{B}T}\cdot \frac{A+Bx+Cx^{2}}{1+Dx+Ex^{2}+Fx^{3}}$$

In Equation (7), x = |J|/k_B_T, and the parameters A–F are constants.^[^ ^48^, ^49^ ^]^ When considering additional contributions to the magnetic susceptibility, including paramagnetic impurities (arising due to lattice defects), the diamagnetism of atomic cores, and potential van Vleck‐type temperature‐independent paramagnetism, the experimental χ_m_ in the HTP can be expressed as Equation (5):

(5) $$\chi_{m}=\chi_{chain}+\frac{C}{T-\theta }+\chi_{0}$$

The magnetic susceptibility data in 270–400 K during the cooling process was fitted using Equation (5) alongside Equation (4). The parameters C, θ and χ_0_, obtained from the magnetic susceptibility fit in the LTP, were used, yielding the parameters |J|/k_B_ = 178.8(9) K with g = 2.16 fixed (Figure S3, Supporting Information). Bonner and Fisher^[^ ^48^ ^]^ demonstrated that the temperature T_max_ at the maximum of magnetic susceptibility and the magnetic exchange constant |J| within an S = ½ Heisenberg AFM regular linear chain follow the relationship: k_B_T_max_/|J| = 1.282. In the HTP of **1**, k_B_T_max_/|J| ≈ 285/178.8 = 1.59, which is slightly larger than the value calculated by Bonner and Fisher.

### 2.3 Dielectric Property

**Figure 4:**
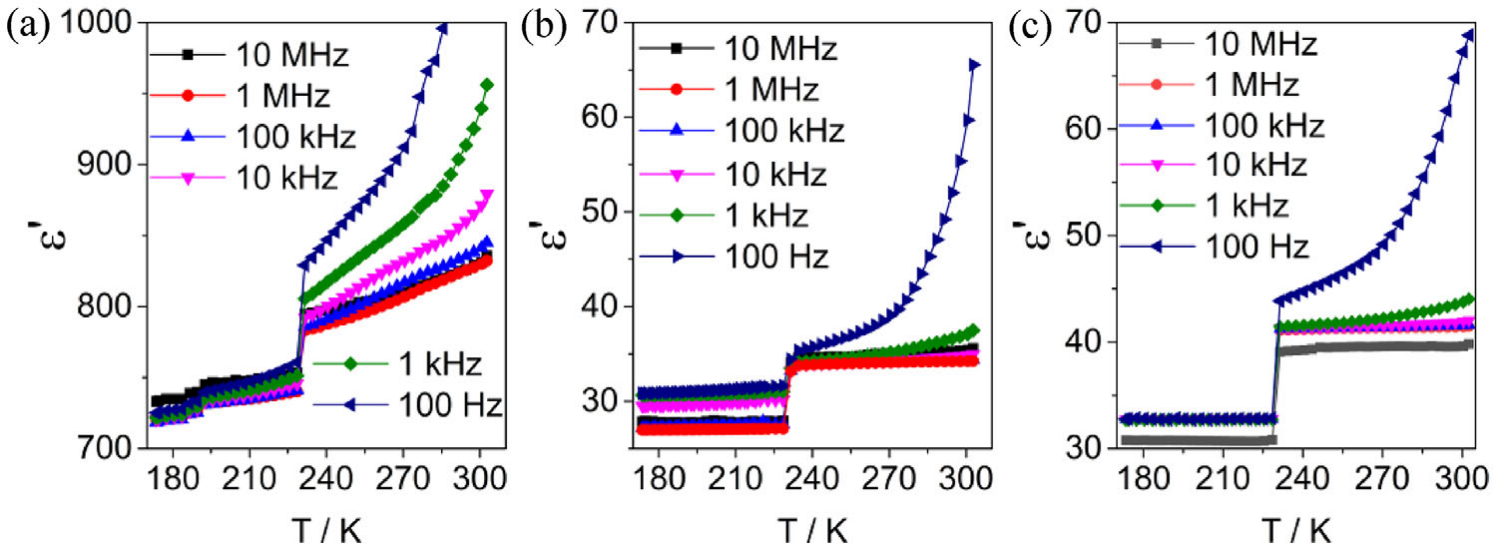
Plots of *ε*′ versus *T* in 173−303 K along the a) *b*‐axis, b) *c*‐axis, and c) *a*‐axis of three individual single crystals of **1** in the frequency range from 100 Hz to 10 MHz during cooling. The crystallographic axes are defined based on the LTP.

The single crystal orientations of **1** were determined using X‐ray diffraction at ambient temperature, as illustrated in Figure S4 (Supporting Information). Dielectric permittivity measurements were performed on six selected single crystals along the b‐, a‐, and c‐axes, which were defined based on the LTP (Figure S5, Supporting Information), and the dielectric spectra are provided in **Figure** **4**, Figures S6 and S7 (Supporting Information). Notably, subsequent measurements for each crystal during cooling and heating were not performed due to crystal breakage.

In the plots of the real part of dielectric permittivity (ε′) versus temperature in 173−303 K (Figure 4; Figures S6 and S7, Supporting Information), a discontinuous change in ε′ is observed at T_↓_ = 228 K during the cooling run for six selected single crystals. This temperature closely aligns with the thermal anomaly temperature observed in the DSC plots and coincides with the temperature range where cell parameters exhibit discontinuous variation, suggesting that the mirror‐symmetry‐broking phase transition in **1** is associated with both a thermal anomaly and a dielectric switching.

In addition to exhibiting a sudden change in dielectric at Tc, **1** demonstrates significant dielectric anisotropy and high‐κ in 173–303 K. Upon cooling within the frequency range of 100 Hz to 10 MHz, the ε′ values range from 719 to 760 along the b‐axis, 27 to 32 along the c‐axis, and 31 to 33 along the a‐axis in the LTP from 173 to 228 K. High‐κ bis(dithiolene)metalate compounds have been reported; however, their dielectric permittivity are less than 20,^[^ ^50^, ^51^, ^52^ ^]^ significantly lower than the values observed in **1**. In the HTP from 232 to 303 K, the ε′ values range from 786 to 996 along the b‐axis, 34 to 42 along the c‐axis, and 39 to 53 along the a‐axis. The significant dielectric anisotropy is attributed to the pronounced crystal structure anisotropy. Similar phenomena have been observed in homochiral trinuclear nickel(II)^[^ ^22^ ^]^ and olefin copper(I) coordination compounds,^[^ ^23^ ^]^ homochiral laminar europium MOFs,^[^ ^24^ ^]^ and polar copper phosphates.^[^ ^25^ ^]^

The dielectric permittivity (ε_r_) of a matter is directly related to its polarization (P), which refers to the displacement of charges within a matter when exposed to an electric field. The relationship between ε_r_ and P depends on various polarization mechanisms, including electronic, ionic, and dipolar contributions. The total polarization P in a matter is the sum of these polarization mechanisms:

(6) $$P=P_{electron}+P_{ion}+P_{dipolar}$$

The dielectric permittivity ε_r_ is related to P as:

(7) $$\varepsilon_{r}=1+\frac{P}{\varepsilon_{0}E}$$

In Equation (7), ε_0_ is the permittivity of free space and E is the applied electric field.

The total polarization P in **1** arises from the electron polarization of the anion and possible displacement polarization of cation. Even though both the anion and cation possess no permanent dipole due to their symmetry, the anion has a π‐electron delocalized on the molecule skeleton, which allows the electrons to be displacible under the influence of an external electric field.

On the other hand, the ion displacement polarization is strongly dependent on the free volume within the lattice, which can be estimated by the Kitaigorodskii packing index (K.P.I.). In both the HTP and LTP, the K.P.I. values were calculated for **1** using PLATON,^[^ ^53^ ^]^ showing a value of 63.6% at 293 K in the HTP versus 69.1% at 150 K in the LTP, which fall below and within the typical range of 65%–77% observed in the ordered molecular crystals. These findings suggest the presence of obvious free volume in the lattice of **1** especially in the HTP. As a result, the ion displacement polarization is feasible under an applied electric field in the HTP.

**Figure 5:**
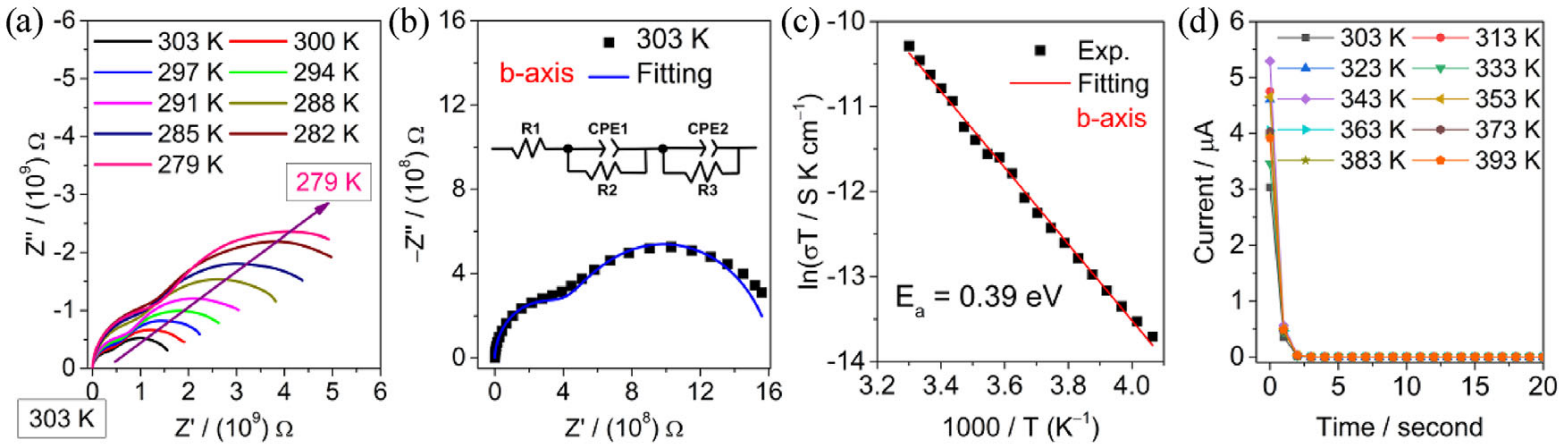
a) Typical impedance spectra at selected temperatures, b) the fitting plot of the impedance spectrum at 303 K using the Z_view_ software with an equivalent circuit (inset), c) experimental and fitted plots ln(*σT*) versus 1000/*T* in 231−303 K, d) Current versus time plot at a voltage of 50 mV in 303−393 K for single crystal of **1** along *b*‐axis. Herein, all axes refer to the LTP monoclinic structure.

To further understand the origin of high‐κ, the impedance spectra of **1** were analyzed. Nyquist plots of Z′ versus Z′′ are nearly linear below 231 K, indicating very low conduction. In this case, the data fitting exhibits a larger error. Consequently, the impedance spectra were analyzed in 231–303 K. **Figures** **5a** and S8 (Supporting Information) show the impedance spectra recorded with the electric field parallel to the b‐axis, while those along the a‐axis and c‐axis are depicted in Figures S9 and S10 (Supporting Information).

Two distinct arcs are observed in all impedance spectra at temperatures above 231 K, corresponding to the resistances of the bulk and grain boundary, respectively, with the former being much smaller than the latter. The conductivity (σ) at a given temperature (T) was determined by fitting its impedance spectrum using the Z_view_ software (Scribner Associates, Inc.) and an equivalent circuit (Figure 5b). The *σ* values were obtained from single crystals with the measuring field along the *b*‐, *c*‐, and *a*‐axes (defined based on LTP), and the activation energy (*E*_a_) was calculated using Arrhenius equation:

(8) $$\ln\left(\sigma T\right)=\mathrm{lnA}\;\frac{E_{a}}{k_{B}T}$$

In Equation (8), the *σ*, *A*, *E*_a_, and k_B_ represent the conductivity, pre‐exponential factor, carrier transport activation energy, and Boltzmann constant, respectively. The best fit was performed for ln(*σT*) versus 1000/*T*, yielding *E*_a_ values of 0.39 eV for the impedance spectra measured with the electric field parallel to the crystallographic *b*‐, *c*‐, and *a*‐axes (Figure 5c; Figure S11, Supporting Information). The conductivity along the *b*‐axis is significantly higher than those along the *c*‐ and *a*‐axes, and this may result in the dielectric permittivity being much higher along‐*b* axis than those along *c*‐ and *a*‐axes.

To differentiate the contributions of electronic and ionic conduction, chronocurrent were measured on two distinct crystals along the b‐axis and approximately c‐axis, respectively. The current–time curves are shown in Figure 5d, Figure S12 (Supporting Information) for crystals of **1** in 303–393 K. An abrupt decrease in current was observed during the initial ≈3 s, from ≈5 to 10^−4^ µA, followed by stabilization at the 10^−4^ µA level. The initial sharp decline in current is attributed to the ionic displacement polarization contribution, while the stabilized current corresponds to the electronic conduction component. These results indicate that electronic conduction is considerably weaker than ion polarization contribution and can be deemed negligible in comparison.

Ion migration can result in dielectric relaxation, a phenomenon that occurs when the movement of ions in response to an applied electric field leads to a delayed polarization response. Temperature‐dependent dielectric modulus plots (Figure S13, Supporting Information) reveal that dielectric relaxations are evident in the HTP but are not observed in the LTP. These findings unveil **1** showing significant ion displacement polarization or migration in the HTP, while absent in the LTP. The activation energies for dielectric relaxation in the HTP are estimated to be 0.34, 0.40, and 0.36 eV based on the analysis of electric modulus along the crystallographic b‐, c‐, and a‐axes, respectively. These E_a_ values are comparable to the activation energies for ion conduction along the corresponding crystal axes.

Based on the above analysis, we conclude that in the HTP, the high‐κ behavior of 1 is primarily attributed to the combined effects of ion displacement polarization and the electron polarization of delocalized π‐electrons on the [Ni(mnt)₂]⁻ anions. In contrast, in the LTP, where ion migration is suppressed, the dielectric response is predominantly governed by the electronic contribution. The dielectric anisotropy arises due to the pronounced structure anisotropy of the crystal. Notably, in the HTP, the b‐axis, which is the shortest crystallographic axis, is aligned parallel to the anion stacks. This alignment results in the highest concentration of polarons along the b‐axis, leading to the highest dielectric permittivity compared to the other crystallographic axes.

Dielectric switching, coupled with magnetic bistability and spontaneous strain in molecule‐based materials, has attracted significant attention due to its potential applications in sensors, information processing, and storage. To the best of our knowledge, examples of compounds exhibiting both dielectric switching and magnetic bistability are exceedingly rare.^[^ ^3^, ^10^, ^18^, ^19^, ^20^, ^21^ ^]^ Compound **1** is the first example to integrate spontaneous strain, magnetic bistability, dielectric switching, and high‐κ functionality.

## 3 Conclusion

In this study, we conduct the study on a 1D S = ½ Heisenberg AFM linear chain compound, which undergoes a novel paraelastic‐ferroelastic phase transition, coupled with magnetic bistability, switchable dielectric and anisotropic high‐κ properties. The symmetry‐breaking structure phase transition is driven by spin‐lattice interactions. The high‐κ is attributed to both a barrier layer capacitor effect, arising due to cation displacement polarization, and the electron polarization of anions with delocalized π‐electrons. These findings unveil the potential for designing multifunctional molecule‐based materials with novel properties suitable for future device applications.

## 4 Experimental Section

### Chemicals and Materials

The starting materials, Na_2_mnt^[^ ^54^ ^]^ and (Et_3_MeN)Cl,^[^ ^55^ ^]^ were synthesized following the reported method in the literatures. (Et_3_MeN)_2_[Ni(mnt)_2_] was prepared according to a similar procedure as used for the preparation of [4′‐NO_2_‐BzPy]_2_[Ni(mnt)_2_] (where 4′‐NO_2_‐BzPy^+^ = 4′‐nitrobenzyl‐1‐pyridinium).^[^ ^56^ ^]^

### Preparation of 1

A MeOH solution (20 cm^3^) of I_2_ (150 mg, 0.59 mmol) was slowly added to a MeCN solution (10 cm^3^) of (Et_3_MeN)_2_[Ni(mnt)_2_] (572 mg, 1.0 mmol), the mixture was allowed standing overnight after stirred for 25 min. The black powdered sample formed was filtered off, washed with MeOH and dried in vacuum. Yield ≈84% (based on (Et_3_MeN)_2_[Ni(mnt)_2_]). Anal. Calc. for C_15_H_18_N_5_NiS_4_: C, 39.57; H, 3.98; N, 15.38%. Found: C, 39.27; H, 3.94; N, 14.95%. The formation of compound **1** was confirmed by the Infrared (IR) spectrum (Figure S1, Supporting Information). IR spectrum (KBr pellet, cm^−1^) and the assignments for the listed bands:^[^ ^57^ ^]^ 2989(m), 2852(m) attributed to the ν_C‐H_ of the alkyl chains; 2208(versus) and 2157(sh) assigned to the ν_C≡N_ of the mnt^2−^ ligands; 1633(s) 1486(versus), and 1418(s) attributed to the ring stretching vibration of the pyridyl and phenyl rings in the cation; 1459(s) arose from the ν_C = C_ of the mnt^2−^ ligands; 718(m), 668(m), 503(s) for π_C‐CN_ and 806(m) attributed to ν_C‐S_ of mnt^2−^ ligands.

The single crystal of compound **1** suitable for single crystal X‐ray diffraction can be obtained by slow evaporation of the saturated acetonitrile of the above powdered sample at ambient temperature for 7−10 days. The phase purity of the crystals was inspected by means of powder X‐ray diffraction technique, the experimental diffraction patterns.

CCDC 2406978(293 K), 2406979(270 K), 2406980(250 K), 2406981(150 K), 2406982(125 K), 2406983(100 K) contains the supplementary crystallographic data for this paper. These data can be obtained free of charge from The Cambridge Crystallographic Data Centre via http://www.ccdc.cam.ac.uk/data_request/cif.]

## Conflict of Interest

The authors declare no conflict of interest.

## Supporting information



Supporting Information

## Data Availability

The data that support the findings of this study are available in the supplementary material of this article.
