# Supplementary material for: Spontaneous Strain‐Spin Transition Coupling Molecular Crystal with Thermal Magnetic Memory Effect, Anisotropic High‐κ and Switchable Dielectric Permittivity
Source: Adv Sci (Weinh). 2025 Jun 4;12(33):e01925. doi: 10.1002/advs.202501925 (PMC12412558; doi:10.1002/advs.202501925)
Supplement: Supplementary file 1 — Supporting Information [file ADVS-12-e01925-s001.docx]

**Supporting Information**

Spontaneous Strain-Spin Transition Coupling Molecular Crystal with Thermal Magnetic Memory Effect, Anisotropic High-κ and Switchable Dielectric Permittivity

Xuan-Rong Chen,*^a,b^ Zhang-Ni He,^a^ Yin Qian,*^a^ Wei Wei,^a^ Zheng-Fang Tian,^c^ Xiao-Ming Ren*^a,d,e^

^a^ State Key Laboratory of Materials-Oriented Chemical Engineering and College of Chemistry and Molecular Engineering, Nanjing Tech University, Nanjing 211816, P. R. China

^b^ School of Chemistry & Environmental Engineering and Instrumental Analysis Center, Yancheng Teachers University, Yancheng 224007, P. R. China

^c^ Hubei Key Laboratory for Processing and Application of Catalytic Materials, Huanggang Normal University, Huanggang, 438000, P. R. China

^d^ College of Materials Science and Engineering, Nanjing Tech University, Nanjing 211816, P. R. China

^e^ State Key Laboratory & Coordination Chemistry Institute, Nanjing University, Nanjing 210023, P. R. China

Tel.: +86 25 58139476

Email: [chenxr@yctu.edu.cn](mailto:chenxr@yctu.edu.cn) (XRC)

yinqian@njtech.edu.cn (YQ)

[xmren@njtech.edu.cn](mailto:xmren@njtech.edu.cn) (XMR)

**Table of Contents**

**Physical measurements**

**X-ray crystallography**

**Table S1:** Crystallographic data and refinement parameter for **1** at 293, 270, 250, 150, 125 and 100 K

**Table S2:** Bond lengths and bond angles in anions and cations in HTP and LTP of **1**

**Figure S1**: (a) ORTEP plot with non-hydrogen atom labeling and the thermal ellipsoid drawn at 20% probability level, (b) parking diagram viewed along b-axis, (c) regular anion stack for **1** at 293 K in HTP.

**Figure S2**: (a) ORTEP plot with non-hydrogen atom labeling and the thermal ellipsoid drawn at 30% probability level, (b) parking diagram viewed along a-axis, (c) the adjacent Ni…Ni distances of two neighboring [Ni(mnt)_2_]^−^ anions, (d) charge-assisted H-bonds between H atoms in the cations and N atoms in the CN groups in the anions (with the symmetry codes: #1 = -*x*, -0.5+*y*, -0.5-*z*; #2 = -*x*, -0.5+*y*, 0.5-*z*) for **1** at 150 K in LTP.

**Figure S3**: Fitting data through spin-gap equation in the temperature range of 1.8−208 K in the LTP and 1D-uniform spin chain in the range of 270−400 K in the HTP for **1** in the cooling process.

**Figure S4**: (a) Dimensions for a selected single crystal of **1**. (b) Illustration for the crystal orientation of the single crystal of **1** determined using X-ray diffraction technique.

**Figure S5**: The photographs for the single crystals of **1** used for dielectric measurements along b-axis, a-axis and c-axis, respectively.

**Figure S6**: Plots of ε′ vs. T in 173−303 K and at selected frequencies along the b-axis during cooling.

**Figure S7**: Plots of ε′ vs. T in 173−303 K along the (a) c-axis, and (b) a-axis of three other selected single crystals of **1** in the frequency range of 100 Hz to 10 MHz during cooling.

**Figure S8**: Typical impedance spectra at the selected temperatures in the range 231−303 K for single crystal of **1** performed along b-axis.

**Figure S9**: Typical impedance spectra at the selected temperatures in the range 237-303 K for single crystal of **1** performed along a-axis.

**Figure S10**: Typical impedance spectra at the selected temperatures in the range 237−303 K for single crystal of **1** performed along c-axis.

**Figure S11**: Plot of ln(σT) vs. 1000/T and the fits obtained using Arrhenius equation in the range of 273−303 K for single crystal of **1** performed along a- and c-axes, respectively.

**Figure S12**: Current variation curve against time at a direct current voltage of 50 mV in the temperature range of 303−393 K for two single crystals, and the measurements along (a, b) b-axis, (c, d) c-axis of crystals, respectively.

**Figure S13**: (a−c) Imaginary part of the electric modulus vs frequency at selected temperatures and (d−f) plots of lnτ vs 1000/T of **1** along b-axis, a-axis and c-axis for the relaxations in the different frequency regions.

**Figure S14**: FT-IR spectrum of **1** (the main characteristic vibration bands ν_C≡N_ of the mnt^2-^ ligand (2208 cm^-1^) and ν_C=C_ of the mnt^2-^ ligand (1460 cm^-1^) are indicated).

**Figure S15**: Experimental and simulated powder X-ray diffraction patterns of **1** at room temperature, indicating that the polycrystalline sample of has high phase purity.

**Figure S16**: (a) TG Plots of **1** in 20−800 ºC (b, c) DSC plot of **1** between −174 and 30 ºC.

**References**

**Physical measurements**

Elemental analyses for C, H and N were performed with an Elementar Vario EL III analytic instrument. Powder X-ray diffraction (PXRD) data were collected on a Bruker D8 diffractometer with Cu Kα radiation (λ = 1.5418 Å). FT-IR spectra were recorded on a Bruker Vertex 80 FT-IR (4000-400 cm^-1^) spectrophotometer with KBr pellets. Differential scanning calorimetry (DSC) was carried out on TA Q2000 power-compensation differential scanning calorimeter in the range of 98−293 K (from −175 to 20 °C) for compound **1** and the warming/cooling rate is 10 K·min^-1^ during the thermal cycles. Magnetic susceptibilities were measured on a Quantum Design MPMS-5 superconducting quantum interference device (SQUID) magnetometer over the temperature range of 1.8−400 K. The measurements of temperature and frequency dependent dielectric permittivity and dielectric loss for the powder sample and the single crystal of compound **1** were carried out employing a concept 80 system (Novocontrol, Germany) in the temperature range 123−303 K (from −150 to 30 °C). The powder sample prepared in the form of pellet with 13 mm diameter and 0.4 mm thickness was sandwiched between two parallel copper electrodes and the AC frequencies span from 1 Hz to 10^7^ Hz. A rod-like single crystal, with dimensions 5 mm × 1 mm × 0.8 mm, was selected. The crystal face indexes of one single crystal of **1** were determined using X-ray diffraction (Fig. S6b). In view of the orthorhombic space group in HTP and cell parameter β = 90°, the (010) plane is approximately perpendicular to the b-axis in **1**. Conducting silver paste was coated on two opposite surfaces (1 mm × 0.8 mm) of the single crystal. Gold wires (80 mm diameter) were used to connect copper electrodes to the single crystal surfaces in order to apply ac frequencies of 1–10^7^ Hz. Unless otherwise specified, all crystallographic directions used in anisotropic property measurements refer to LTP, as the measurements were performed before the ferroelastic phase transition.”

**X-ray crystallography**

Single-crystal X-ray diffraction data were collected for compound **1** at 293, 270, 250, 125, 150 K using the graphite-monochromated Mo-Kα radiation (λ = 0.71073 Å) on a CCD area detector (Bruker SMART). Data reductions and absorption corrections were carried out with SAINT and SADABS software packages,^58^ respectively. Structures were solved by the direct method and refined by the full-matrix least-squares procedure on *F^2^* using SHELXL-2018 program.^59^ The non-hydrogen atoms were anisotropically refined using the full-matrix least-square method on *F^2^*. All hydrogen atoms were placed in calculated positions and refined as riding on the parent atoms. The asymmetric units and the packing views were drawn using DIAMOND (Brandenburg and Putz, 2006)^60^ Visual Crystal Structure Information System Software. The crystallographic details about data collection and structural refinement at different temperatures are summarized in Table S1.

**Spontaneous Strain Analysis**

The spontaneous strain components are calculated, based on the lattice parameters. The species with Aizu notation of mmmF2/m has two different orientational states (S_1_ and S_2_), corresponding to the spontaneous strain tensors:

$X_{S}\left( S_{1} \right)=\left( \begin{matrix} 0 & 0 & b \\ 0 & 0 & 0 \\ b & 0 & 0 \end{matrix} \right)$ and $X_{S}\left( S_{2} \right)=\left( \begin{matrix} 0 & 0 & -b \\ 0 & 0 & 0 \\ -b & 0 & 0 \end{matrix} \right)$

Where b = x_13_, and the spontaneous strain x_s_ = $\sqrt{2}$b. The x_13_ can be calculated using the equation below:

$x_{13}=\frac{1}{2}(\frac{c\cdot cos\beta}{c_{0}\cdot sin\beta_{0}}-\frac{a\cdot cos\beta_{0}}{a_{0}\cdot sin\beta_{0}})$ (S1)

In Equation (S1), a, c, β and a_0_, c_0_, β_0_ are the cell parameters of **1** in the LTP (150 K) and HTP (250 K), respectively. The spontaneous strain x_s_ is estimated as 0.0029 for **1**.

**Table S1:** Crystallographic data and refinement parameter for **1** at 293, 270, 250, 150, 125 and 100 K

| Temperature/K | 293(2) | 270(2) | 250(2) | 150(2) | 125(2) | 100(2) |
| --- | --- | --- | --- | --- | --- | --- |
| CCDC numbers | [2406978](https://www.ccdc.cam.ac.uk/mystructures/viewinaccessstructures/367957f9-afb0-ef11-96cd-00505695f620) | [2406979](https://www.ccdc.cam.ac.uk/mystructures/viewinaccessstructures/367957f9-afb0-ef11-96cd-00505695f620) | [2406980](https://www.ccdc.cam.ac.uk/mystructures/viewinaccessstructures/367957f9-afb0-ef11-96cd-00505695f620) | [2406981](https://www.ccdc.cam.ac.uk/mystructures/viewinaccessstructures/367957f9-afb0-ef11-96cd-00505695f620) | [2406982](https://www.ccdc.cam.ac.uk/mystructures/viewinaccessstructures/367957f9-afb0-ef11-96cd-00505695f620) | [2406983](https://www.ccdc.cam.ac.uk/mystructures/viewinaccessstructures/367957f9-afb0-ef11-96cd-00505695f620) |
| Chemical_formua | C_15_H_18_N_5_NiS_4_ | C_15_H_18_N_5_NiS_4_ | C_15_H_18_N_5_NiS_4_ | C_15_H_18_N_5_NiS_4_ | C_15_H_18_N_5_NiS_4_ | C_15_H_18_N_5_NiS_4_ |
| Formula weight | 455.29 | 455.29 | 455.29 | 455.29 | 455.29 | 455.29 |
| Wavelength (Å ) | 0.71073 | 0.71073 | 0.71073 | 0.71073 | 0.71073 | 0.71073 |
| Crystal system | Orthorhombic | Orthorhombic | Orthorhombic | Monoclinic | Monoclinic | Monoclinic |
| Space group | *Pnma* | *Pnma* | *Pnma* | *P*2_1_*/c* | *P*2_1_*/c* | *P*2_1_*/c* |
| *a* (Å) | 15.856(2) | 15.810(3) | 15.823(3) | 7.0601(11) | 7.0254(18) | 6.9998(15) |
| *b* (Å) | 7.0531(10) | 7.0205(11) | 7.0349(13) | 19.381(3) | 19.345(4) | 19.355(4) |
| *c* (Å) | 18.867(3) | 18.840(3) | 18.830(4) | 14.598(2) | 14.574(4) | 14.554(3) |
| α (°) | 90 | 90 | 90 | 90 | 90 | 90 |
| β (°) | 90 | 90 | 90 | 90.253(5) | 90.164(7) | 90.105(6) |
| γ (°) | 90 | 90 | 90 | 90 | 90 | 90 |
| V(Å^3^) / Z | 2110.0(5)/4 | 2084.7(6)/4 | 2097.0(6)/4 | 1997.5(5)/4 | 1980.7(8) /4 | 1971.7(7) /4 |
| ρ (g⋅cm^-3^) | 1.433 | 1.348 | 1.341 | 1.514 | 1.527 | 1.534 |
| F(000) | 940 | 876 | 876 | 940 | 940 | 940 |
| Abs. coeff. (mm^-1^) | 1.323 | 1.237 | 1.230 | 1.398 | 1.410 | 1.416 |
| θ Ranges (data collection°) | 1.68-27.56 | 1.68-27.51 | 1.68-27.7 | 1.05-27.71 | 1.75-26.00 | 1.75-27.59 |
| Index range | -18 ≤ h ≤19 -8 ≤ k ≤ 8  -23 ≤ l ≤ 21 | -20 ≤h≤20  -8 ≤k≤9 -22≤l≤24 | -20 ≤ h ≤ 20 -9 ≤ k ≤ 8  -24 ≤ l ≤ 22 | -9 ≤ h ≤ 9  -24 ≤ k ≤ 22 -19 ≤ l ≤ 16 | -8 ≤ h ≤ 8  -23 ≤ k ≤ 23  0 ≤ l ≤ 17 | -9 ≤ h ≤ 9  -25 ≤ k ≤ 25  0 ≤ l ≤ 18 |
| R_int_ | 0.0702 | 0.0684 | 0.0786 | 0.0687 | 0.0640 | 0.0640 |
| Independent reflections /restraints/parameters | 2246/56/146 | 2572/146/146 | 2642/14/146 | 4529/0/231 | 3858/0/230 | 4422/0/231 |
| Refine method | Full-matrix least-squares on *F^2^* | | | | | |
| Goodness-of-fit on *F*^2^ | 1.081 | 1.033 | 1.057 | 1.081 | 1.012 | 1.027 |
| R_1_, wR_2_ [I>2σ(I)] | R_1_ = 0.0873  wR_2_= 0.2248 | R_1_ = 0.1038  wR_2_= 0.3440 | R_1_ = 0.1347  wR_2_= 0.4176 | R_1_ =0.0590  wR_2_=0.1119 | R_1_ = 0.0454  wR_2_= 0.1302 | R_1_ = 0.0517  wR_2_= 0.1099 |
| R_1_, wR_2_ [all data] | R_1_ = 0.1375  wR_2_= 0.2594 | R_1_ = 0.1711  wR_2_= 0.4051 | R_1_ = 0.1974  wR_2_= 0.4784 | R_1_ =0.0827  wR_2_=0.1271 | R_1_ = 0.0521  wR_2_= 0.1362 | R_1_ = 0.0625  wR_2_= 0.1180 |
| Residual (e⋅nm^-3^) | 1.188/-0.611 | 1.185/ -0.473 | 2.035/ -0.554 | 0.683/-1.119 | 0.482/ -0.705 | 0.714/ -0.655 |

R_1_ = Σ(||F_o_|-|F_c_||)/Σ|F_o_|, wR_2_ = Σw(|F_o_|^2^-|F_c_|^2^)^2^/Σw (|F_o_|^2^)^2^]^1/2^

**Table S2:** Bond lengths and bond angles in anions and cations in HTP and LTP of **1**

| **Bond** | **HTP-293 K** | **HTP-270 K** | **HTP-250 K** | **LTP-150 K** | **LTP-125 K** |
| --- | --- | --- | --- | --- | --- |
| Ni(1)-S(1) | 2.143(5) | 2.135(3) | 2.143(3) | 2.1510(15) | 2.142(6) |
| Ni(1)-S(2) | 2.127(5) | 2.128(3) | 2.136(3) | 2.1457(15) | 2.123(6) |
| Ni(1)-S(3) | 2.139(5) | 2.132(3) | 2.138(3) | 2.1454(16) | 2.174(7) |
| Ni(1)-S(4) | 2.140(6) | 2.146(3) | 2.152(3) | 2.1602(15) | 2.143(6) |
| S(1)-C(2) | 1.718(17) | 1.697(13) | 1.758(12) | 1.711(5) | 1.71(2) |
| S(2)-C(3) | 1.69(2) | 1.699(13) | 1.680(11) | 1.708(5) | 1.72(2) |
| S(3)-C(6) | 1.692(19) | 1.711(12) | 1.778(15) | 1.725(6) | 1.70(3) |
| S(4)-C(7) | 1.66(2) | 1.715(13) | 1.708(12) | 1.735(6) | 1.68(3) |
| C(1)-C(2) | 1.46(3) | 1.461(17) | 1.486(17) | 1.452(7) | 1.48(3) |
| C(2)-C(3) | 1.35(3) | 1.350(15) | 1.310(15) | 1.363(7) | 1.34(4) |
| C(3)-C(4) | 1.47(3) | 1.402(19) | 1.43(2) | 1.443(7) | 1.42(4) |
| C(5)-C(6) | 1.35(4) | 1.43(2) | 1.40(2) | 1.448(8) | 1.46(4) |
| C(6)-C(7) | 1.39(3) | 1.376(15) | 1.351(17) | 1.356(8) | 1.41(3) |
| C(7)-C(8) | 1.44(4) | 1.446(18) | 1.461(18) | 1.441(8) | 1.45(3) |
| N(1)-C(1) | 1.05(4) | 1.140(16) | 1.021(16) | 1.136(7) | 1.12(3) |
| N(2)-C(4) | 1.11(3) | 1.139(18) | 1.18(2) | 1.145(6) | 1.15(4) |
| N(3)-C(5) | 1.24(4) | 1.100(17) | 1.099(19) | 1.126(7) | 1.15(4) |
| N(4)-C(8) | 1.14(4) | 1.096(17) | 1.158(17) | 1.145(8) | 1.14(3) |
| N(5)-C(9) | 1.51(9) | 1.54(4) | 1.454(14) | 1.524(8) | 1.48(4) |
| N(5)-C(12) | 1.62(5) | 1.55(3) | 1.519(13) |  |  |
| N(5)-C(13) | 1.14(19) | 1.35(3) | 1.602(13) |  |  |
| S(2)-Ni(1)-S(3) | 179.2(2) | 179.27(13) | 179.41(12) | 177.57(7) | 171.8(3) |
| S(2)-Ni(1)-S(4) | 87.4(2) | 87.00(12) | 86.94(11) | 88.19(6) | 87.0(2) |
| S(3)-Ni(1)-S(4) | 91.80(18) | 92.27(12) | 92.46(11) | 92.74(6) | 92.4(2) |
| S(2)-Ni(1)-S(1) | 92.76(19) | 92.32(12) | 92.36(12) | 92.61(6) | 92.5(2) |
| S(3)-Ni(1)-S(1) | 88.07(18) | 88.42(11) | 88.23(11) | 86.56(6) | 88.1(3) |
| S(4)-Ni(1)-S(1) | 179.87(19) | 179.32(12) | 179.31(12) | 177.27(7) | 179.1(3) |

(a)


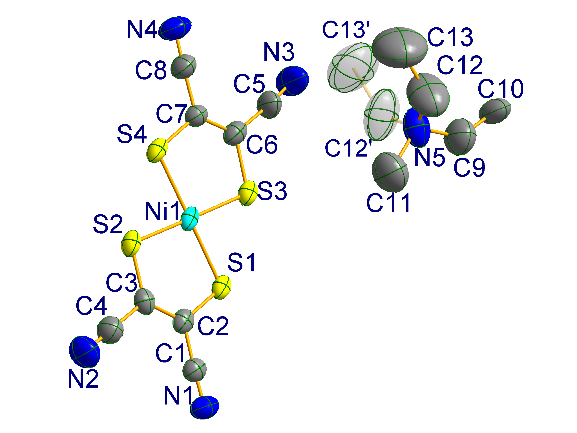


(b)


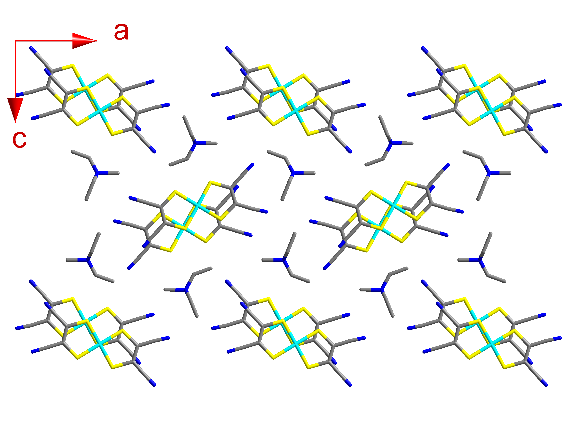

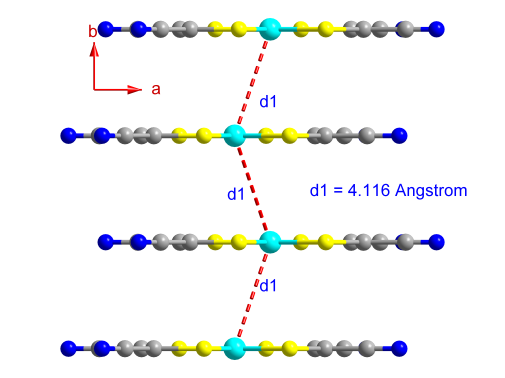


(c)

**Figure S1**: (a) ORTEP plot with non-hydrogen atom labeling and the thermal ellipsoid drawn at 20% probability level, (b) parking diagram viewed along b-axis, (c) regular anion stack for **1** at 293 K in HTP.

(c)

(a)


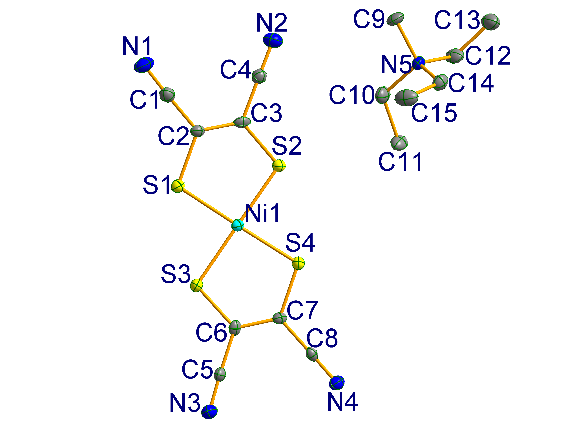

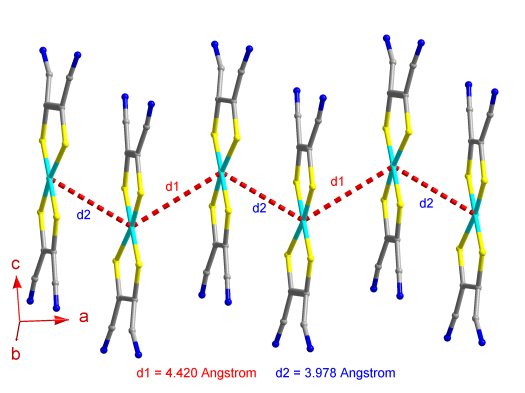


(d)

(b)


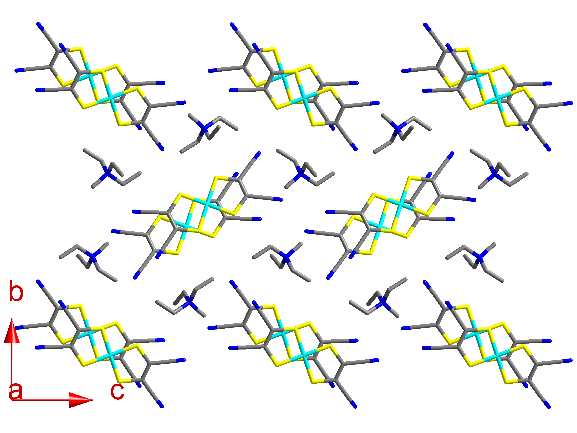

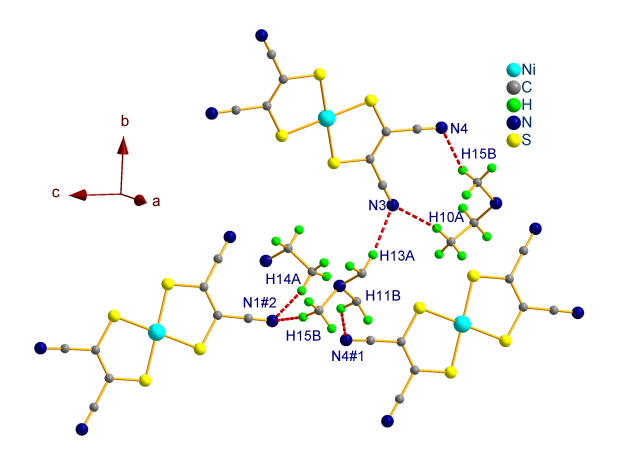


**Figure S2**: (a) ORTEP plot with non-hydrogen atom labeling and the thermal ellipsoid drawn at 30% probability level, (b) parking diagram viewed along a-axis, (c) the adjacent Ni…Ni distances of two neighboring [Ni(mnt)_2_]^−^ anions, (d) charge-assisted H-bonds between H atoms in the cations and N atoms in the CN groups in the anions (with the symmetry codes: #1 = -*x*, -0.5+*y*, -0.5-*z*; #2 = -*x*, -0.5+*y*, 0.5-*z*) for **1** at 150 K in LTP.


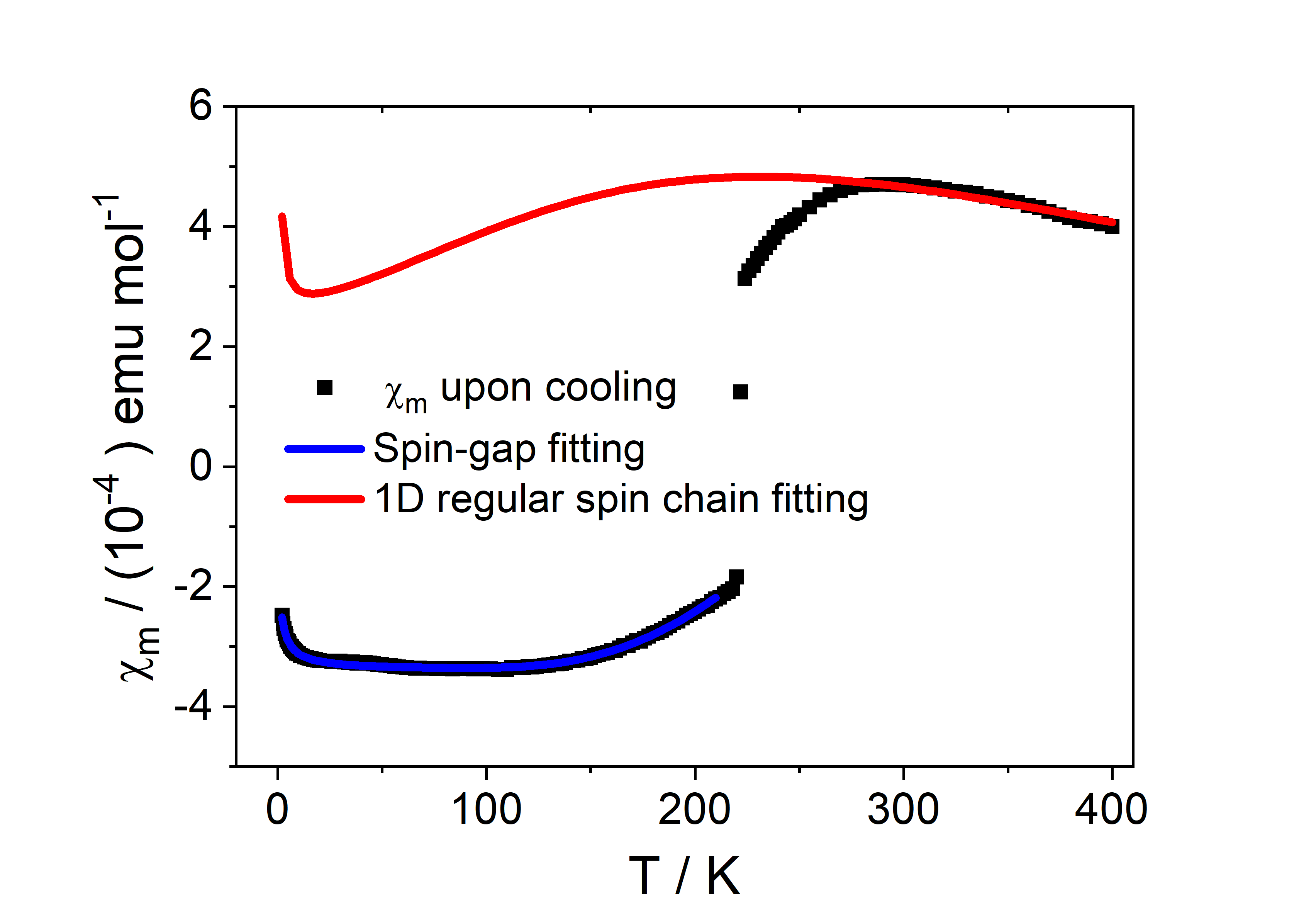


**Figure S3**: Fitting data through spin-gap equation in the temperature range of 1.8−208 K in the LTP and 1D-uniform spin chain in the range of 270−400 K in the HTP for **1** in the cooling process.

(b)

(a)


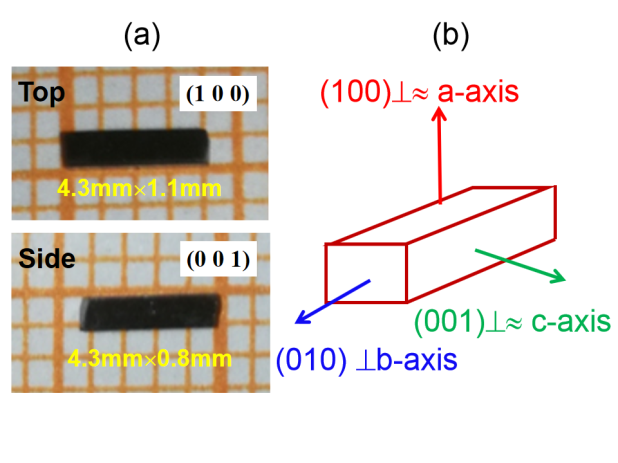


**Figure S4**: (a) Dimensions for a selected single crystal of **1**. (b) Illustration for the orientation of the single crystal of **1** determined using X-ray diffraction technique.


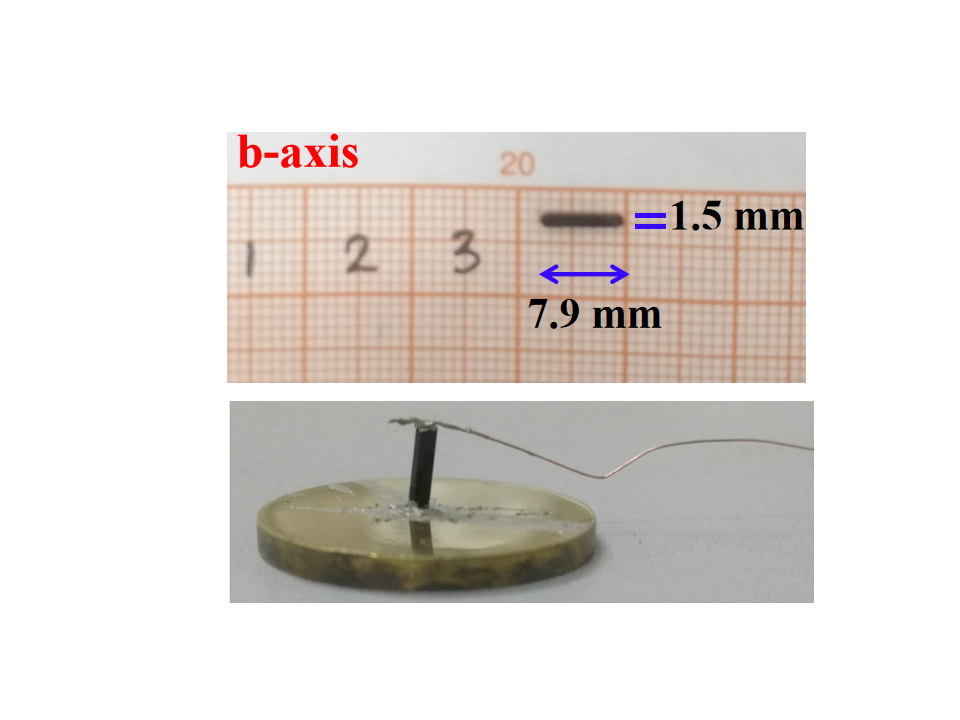

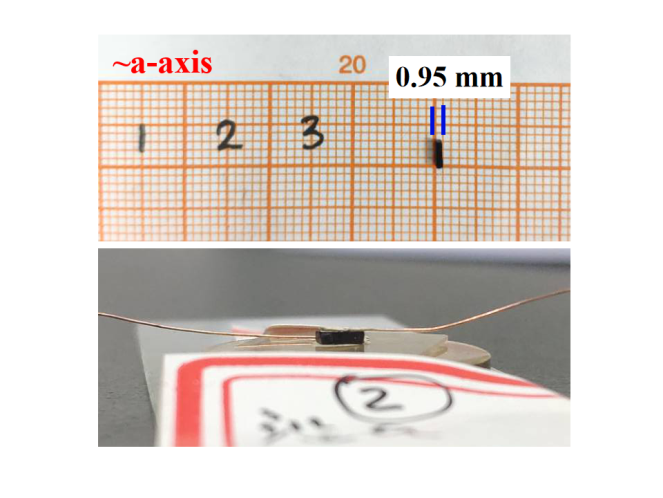


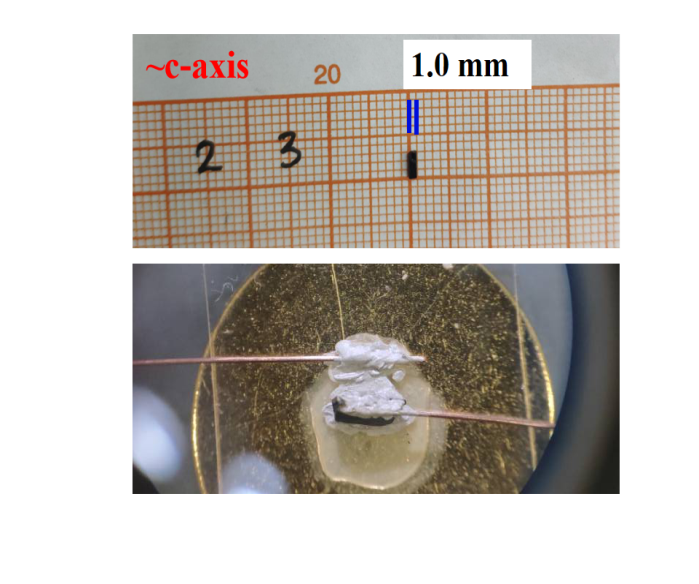


**Figure S5**: The photographs for the single crystals of **1** used for dielectric measurements along b-axis, a-axis and c-axis, respectively.


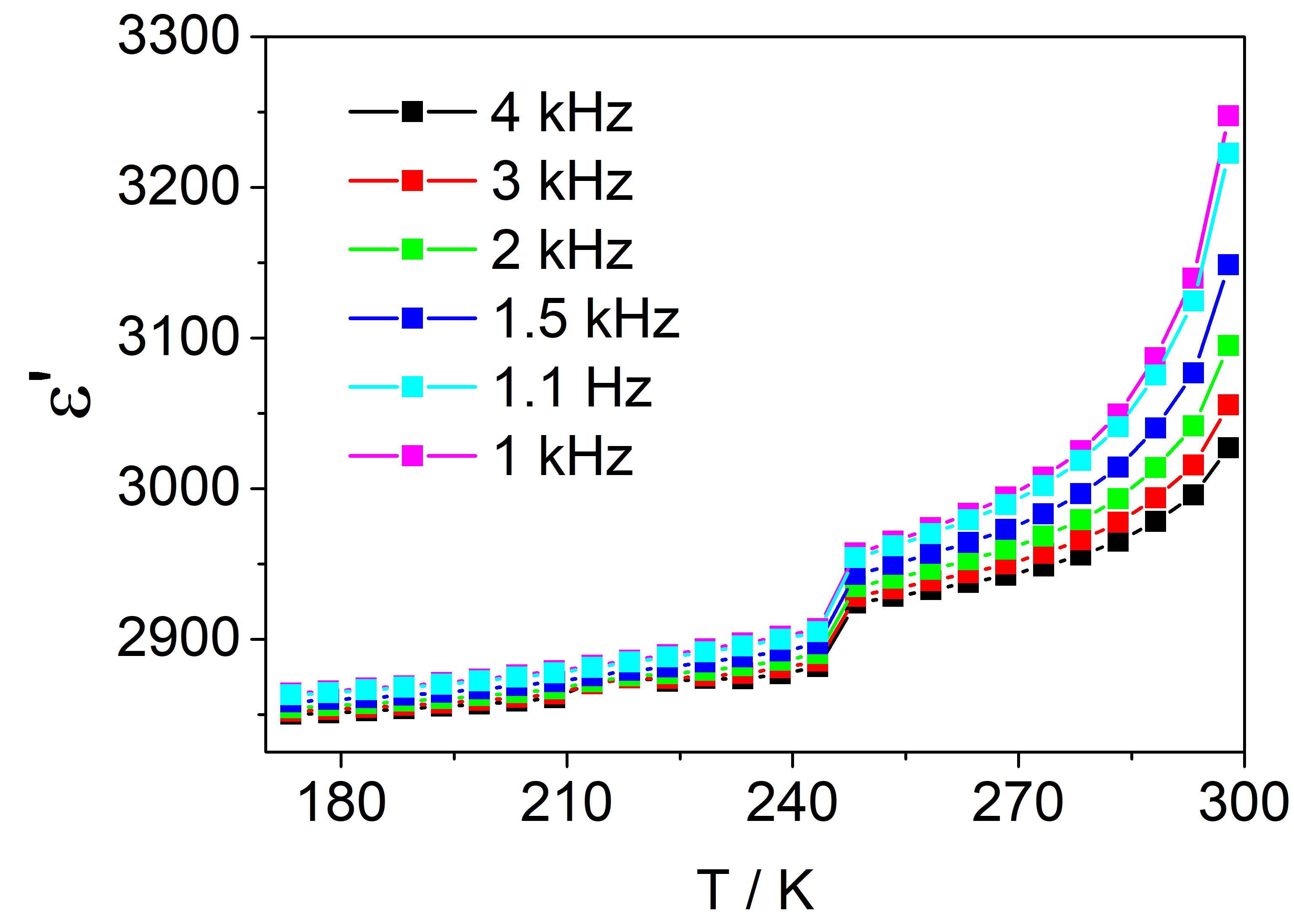


**Figure S6**: Plots of ε′ vs. T in 173−303 K and at selected frequencies along the b-axis during cooling.


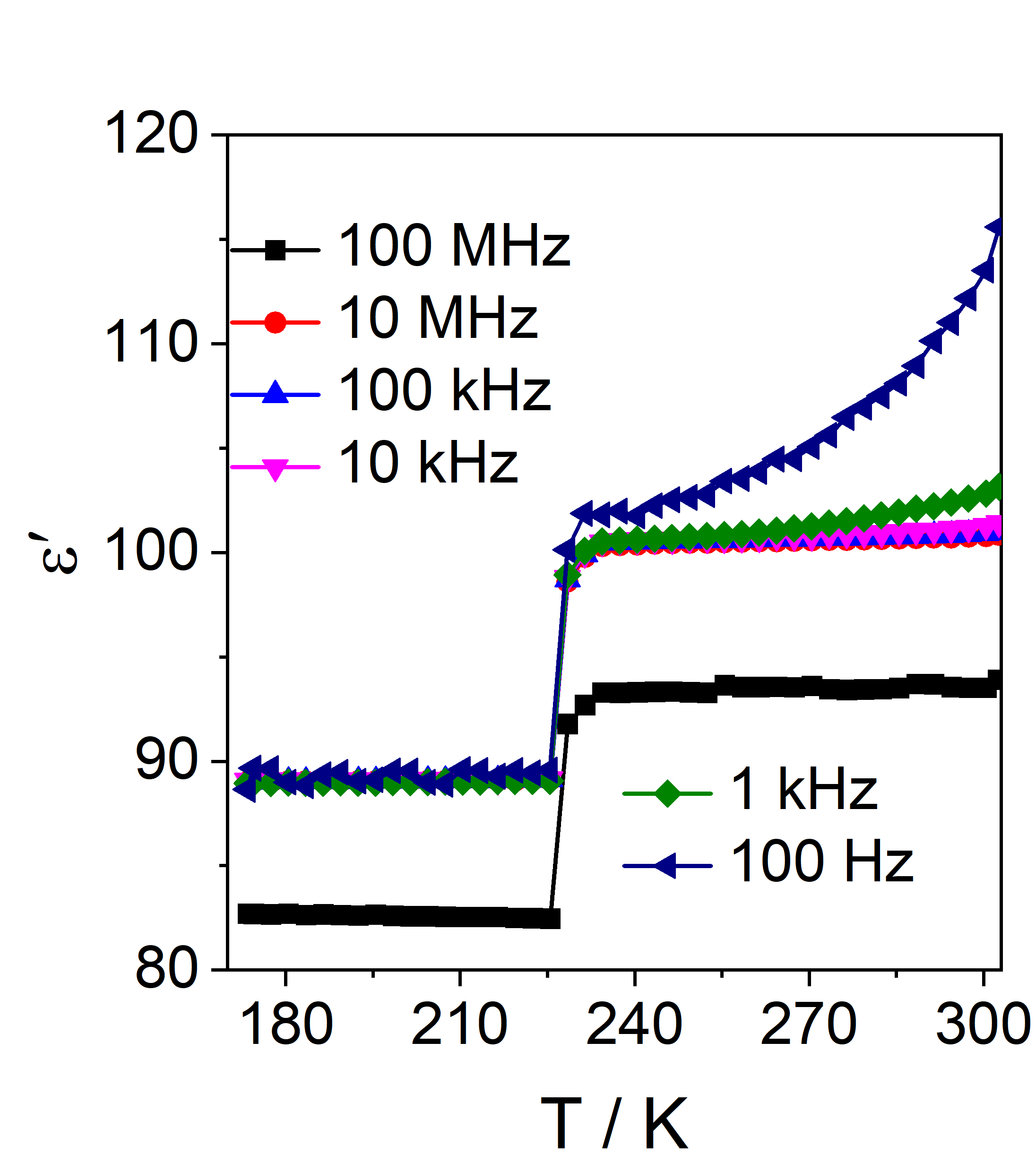

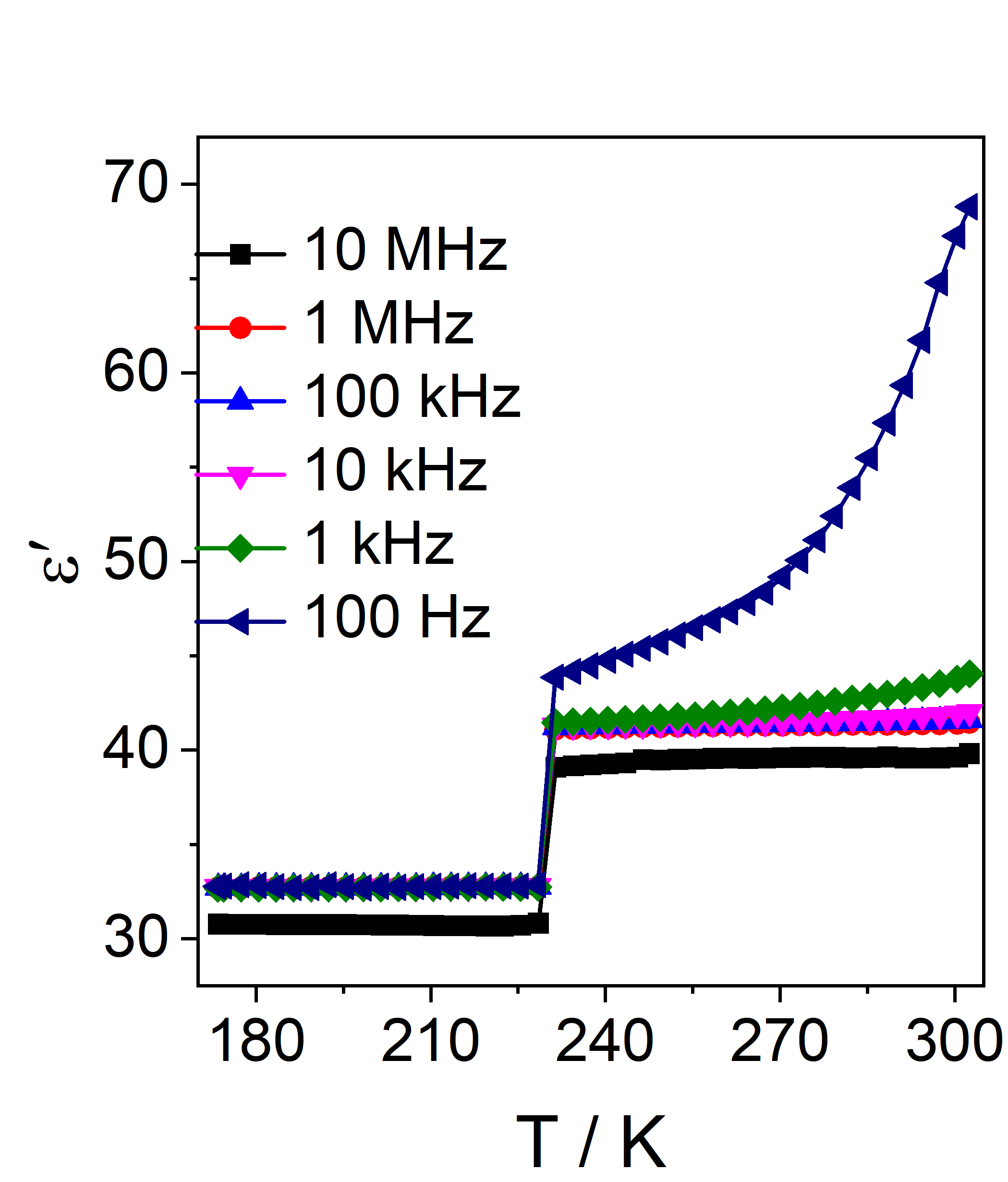


(b)

(a)

**Figure S7**: Plots of ε′ vs. T in 173−303 K along the (a) c-axis, and (b) a-axis of three other selected single crystals of **1** in the frequency range of 100 Hz to 10 MHz during cooling.


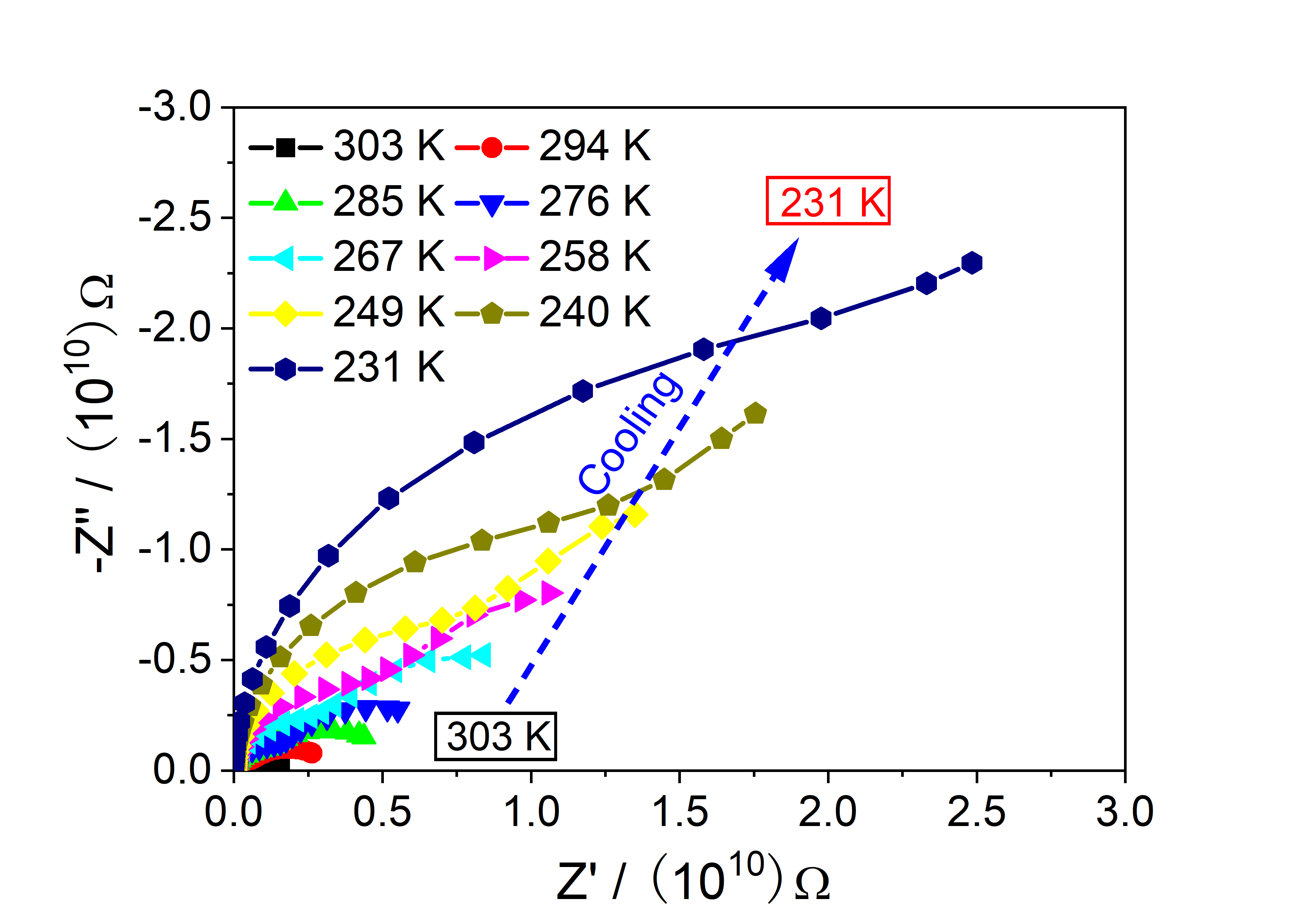


**Figure S8**: Typical impedance spectra at the selected temperatures in the range 231−303 K for single crystal of **1** performed along b-axis.


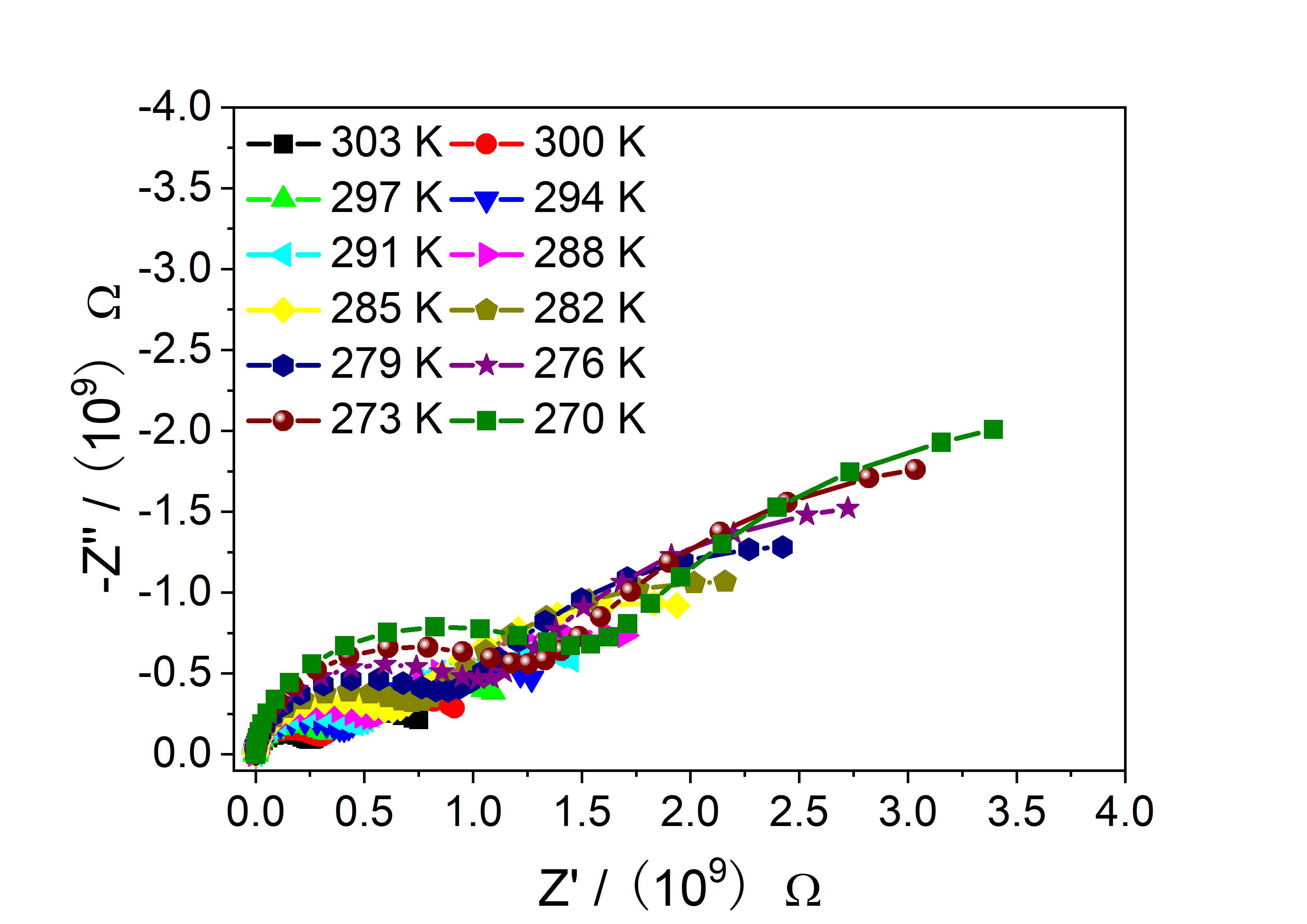

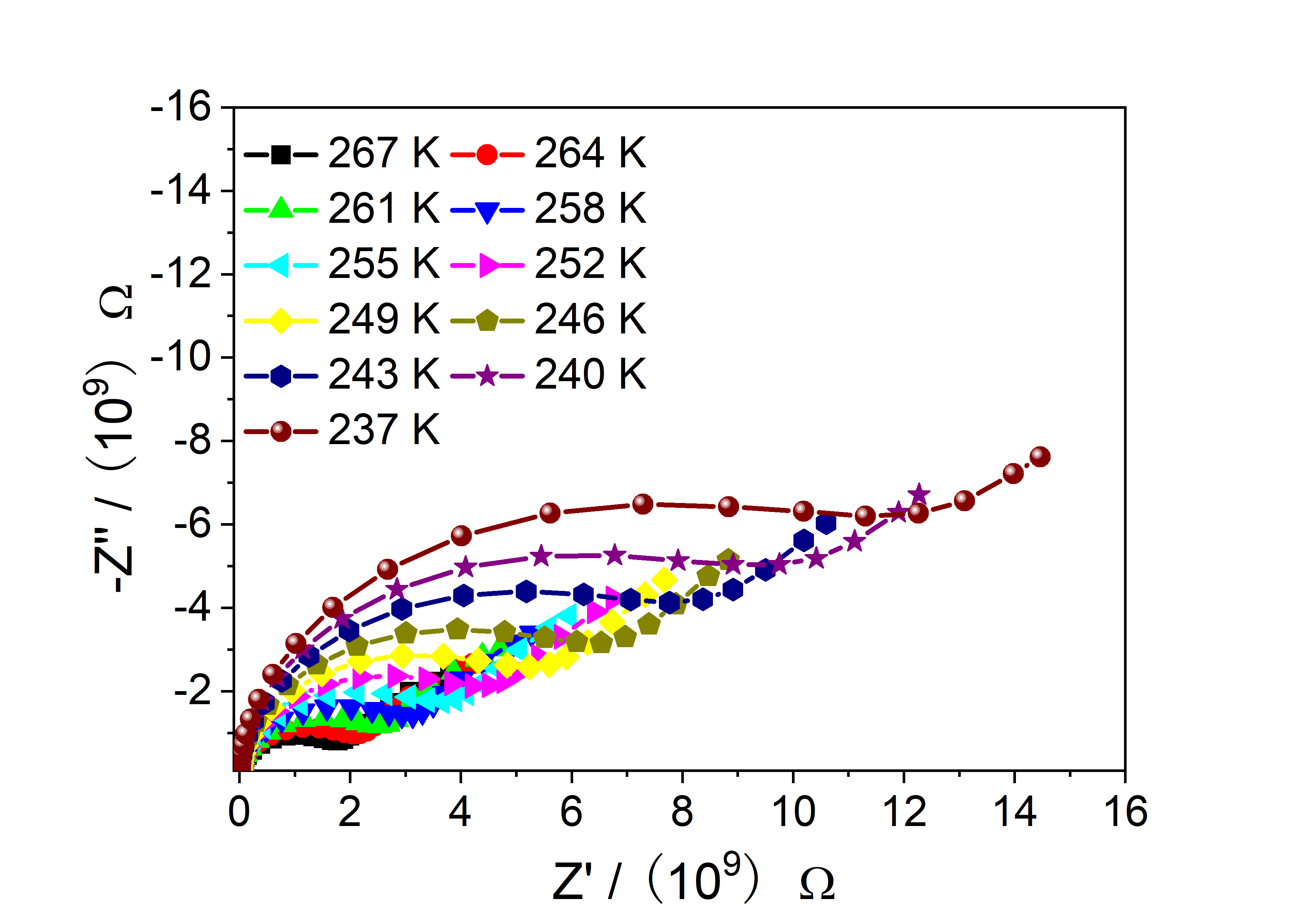


**Figure S9**: Typical impedance spectra at the selected temperatures in the range 237-303 K for single crystal of **1** performed along a-axis.


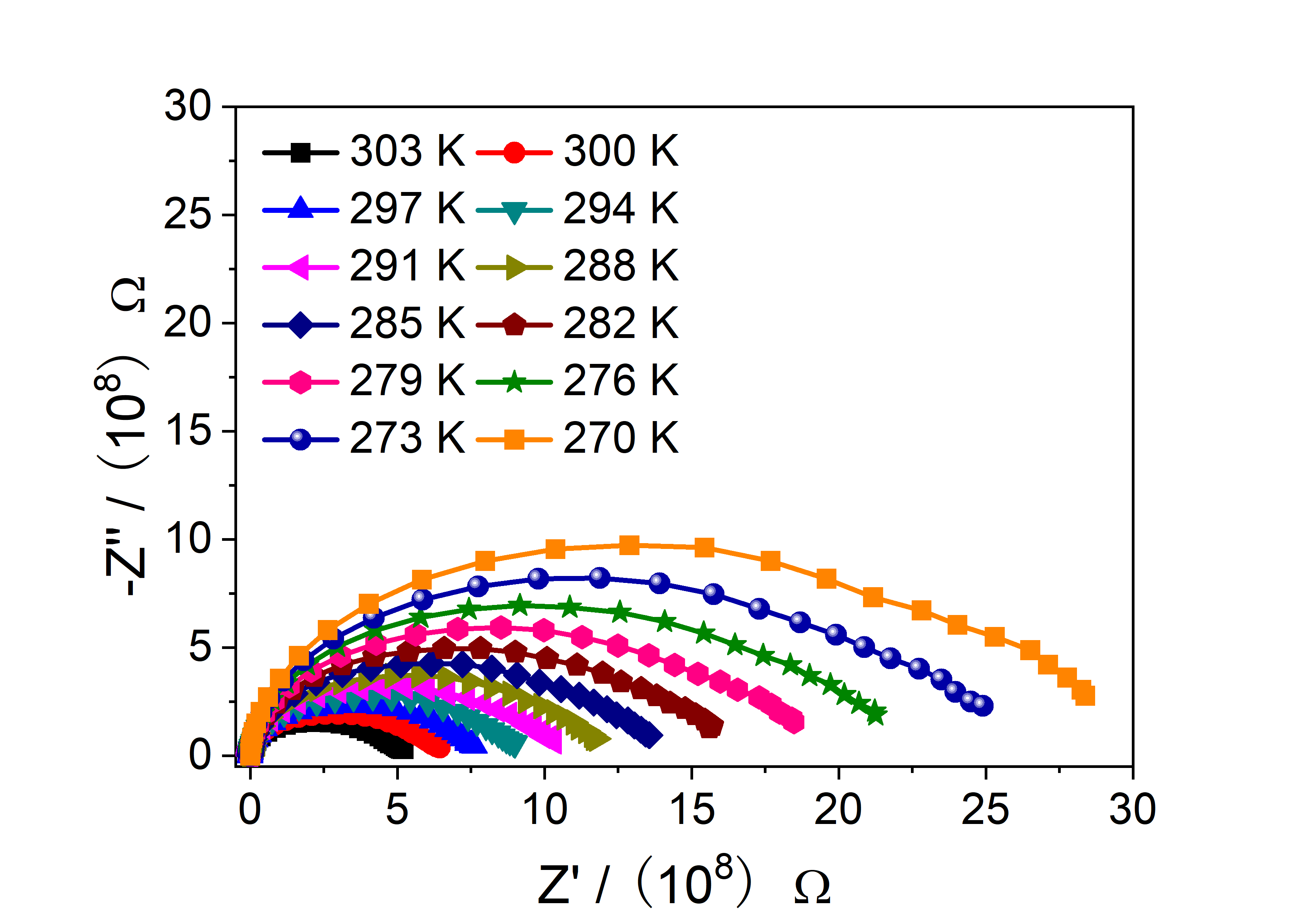

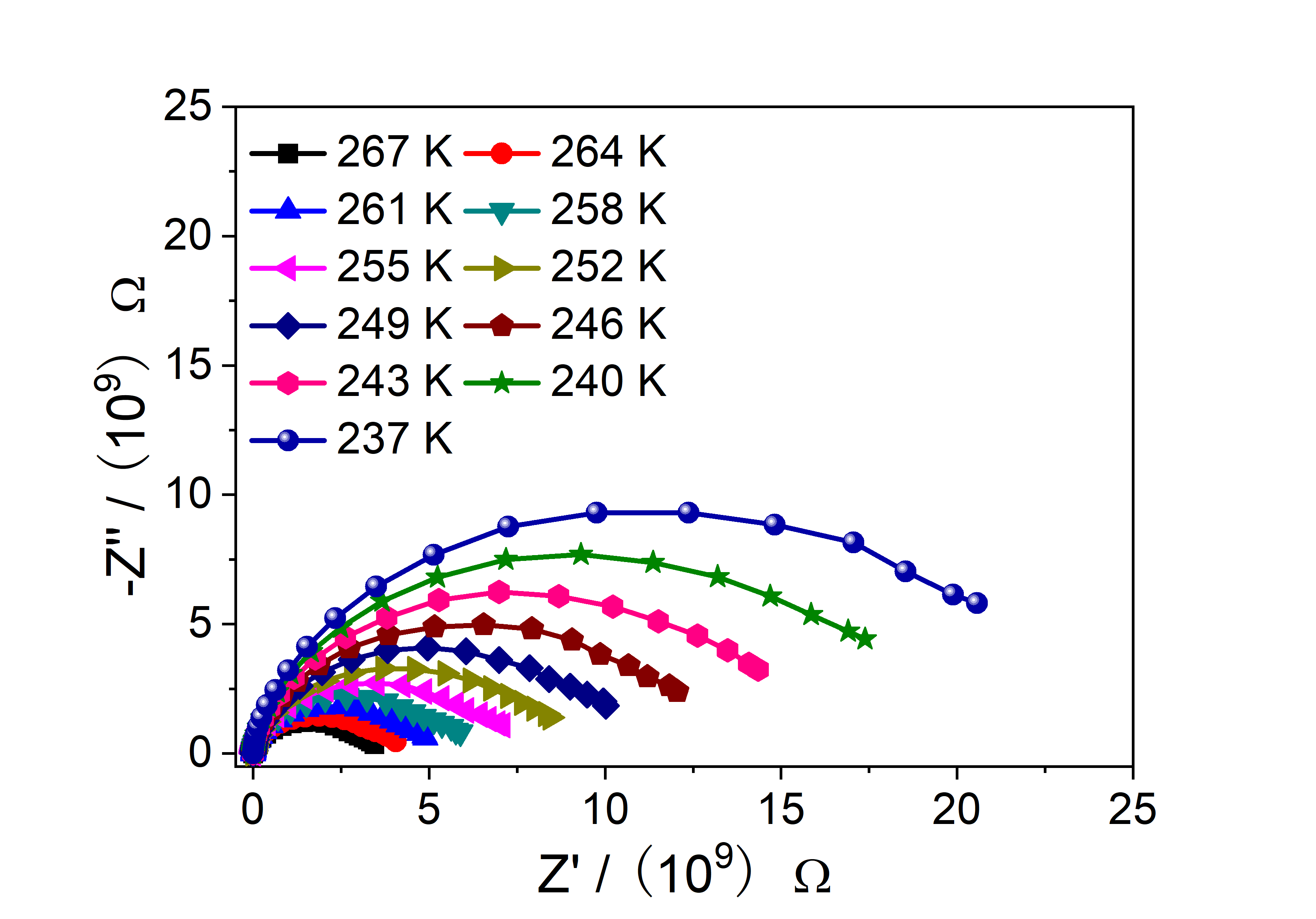


**Figure S10**: Typical impedance spectra at the selected temperatures in the range 237−303 K for single crystal of **1** performed along c-axis.


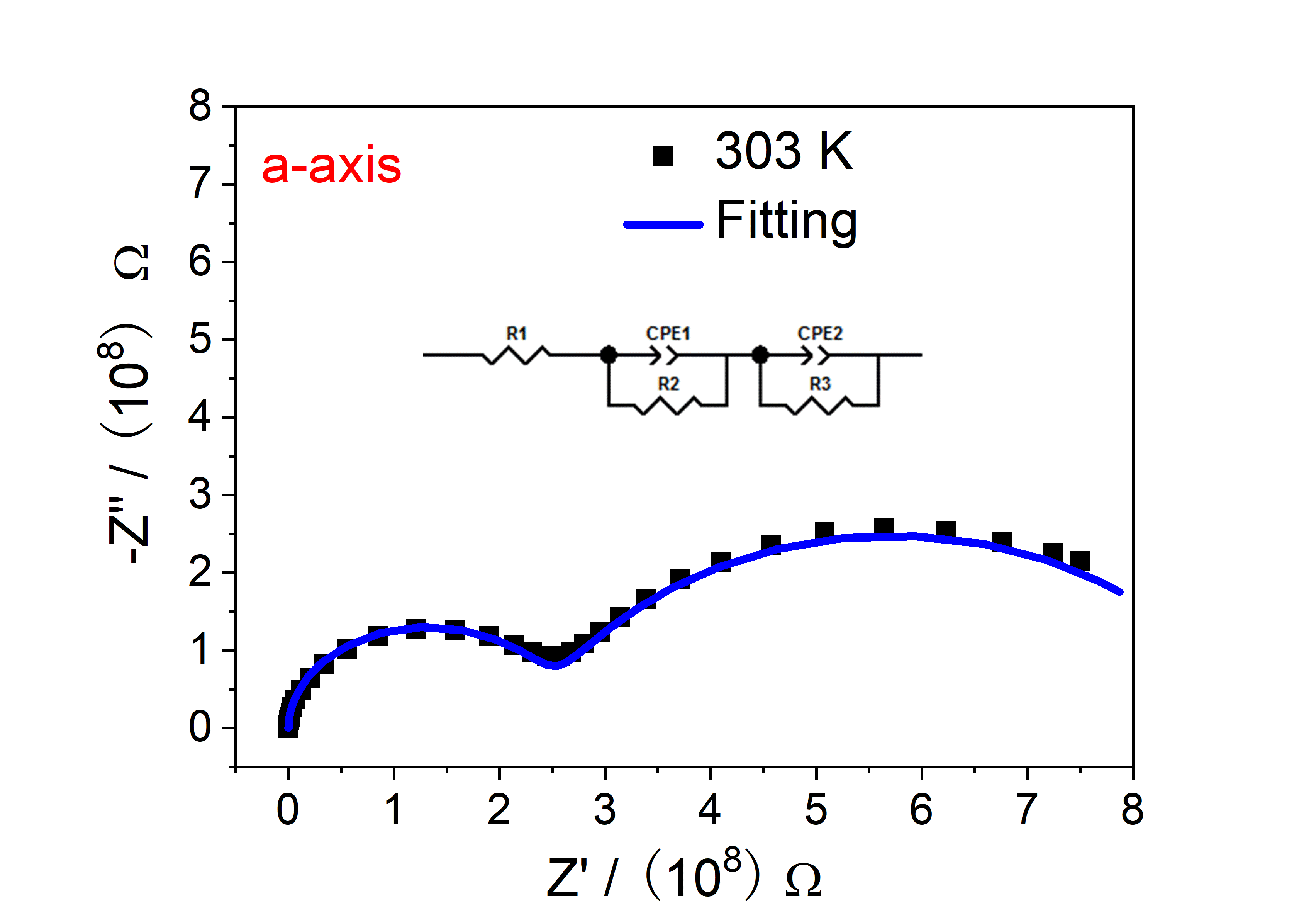

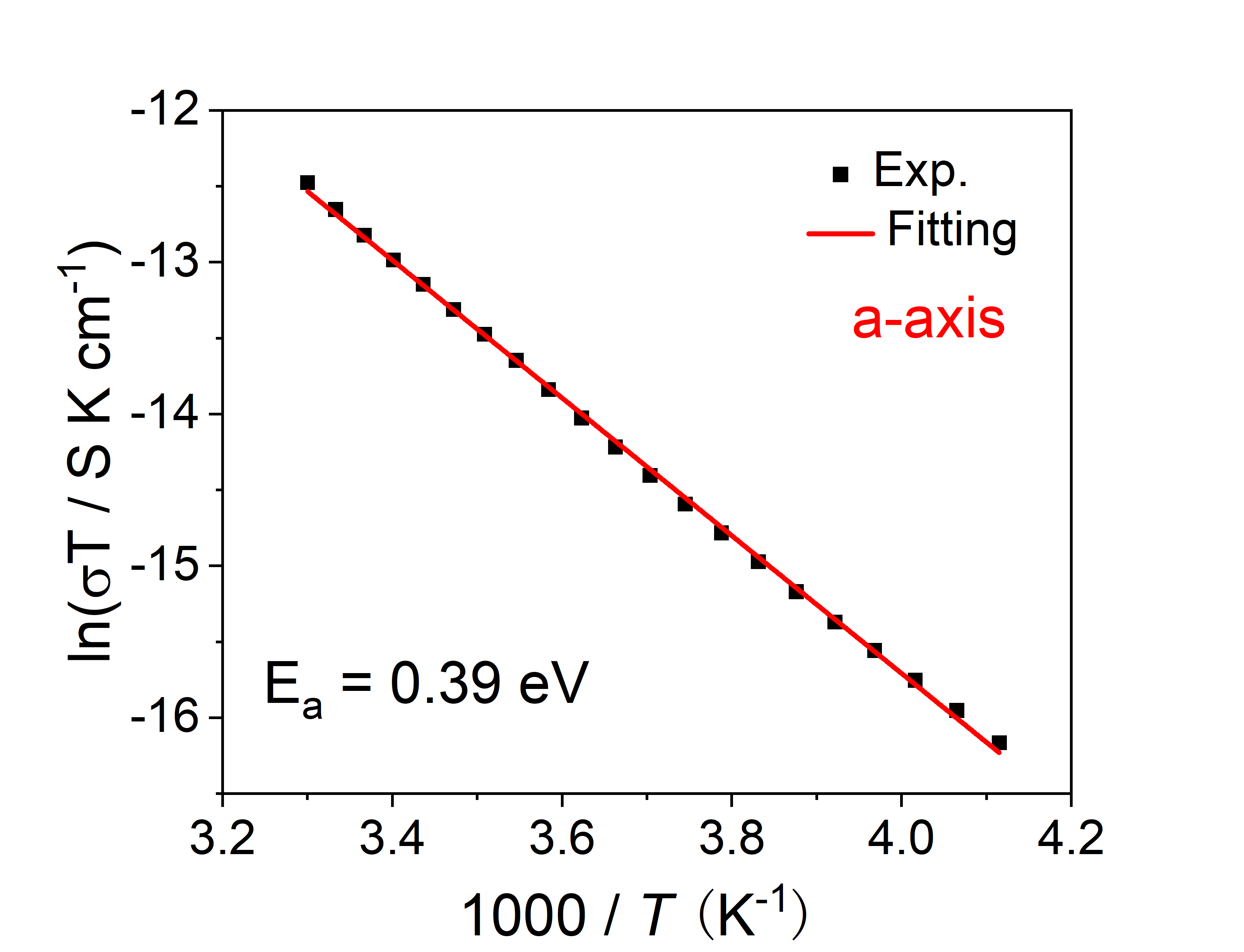


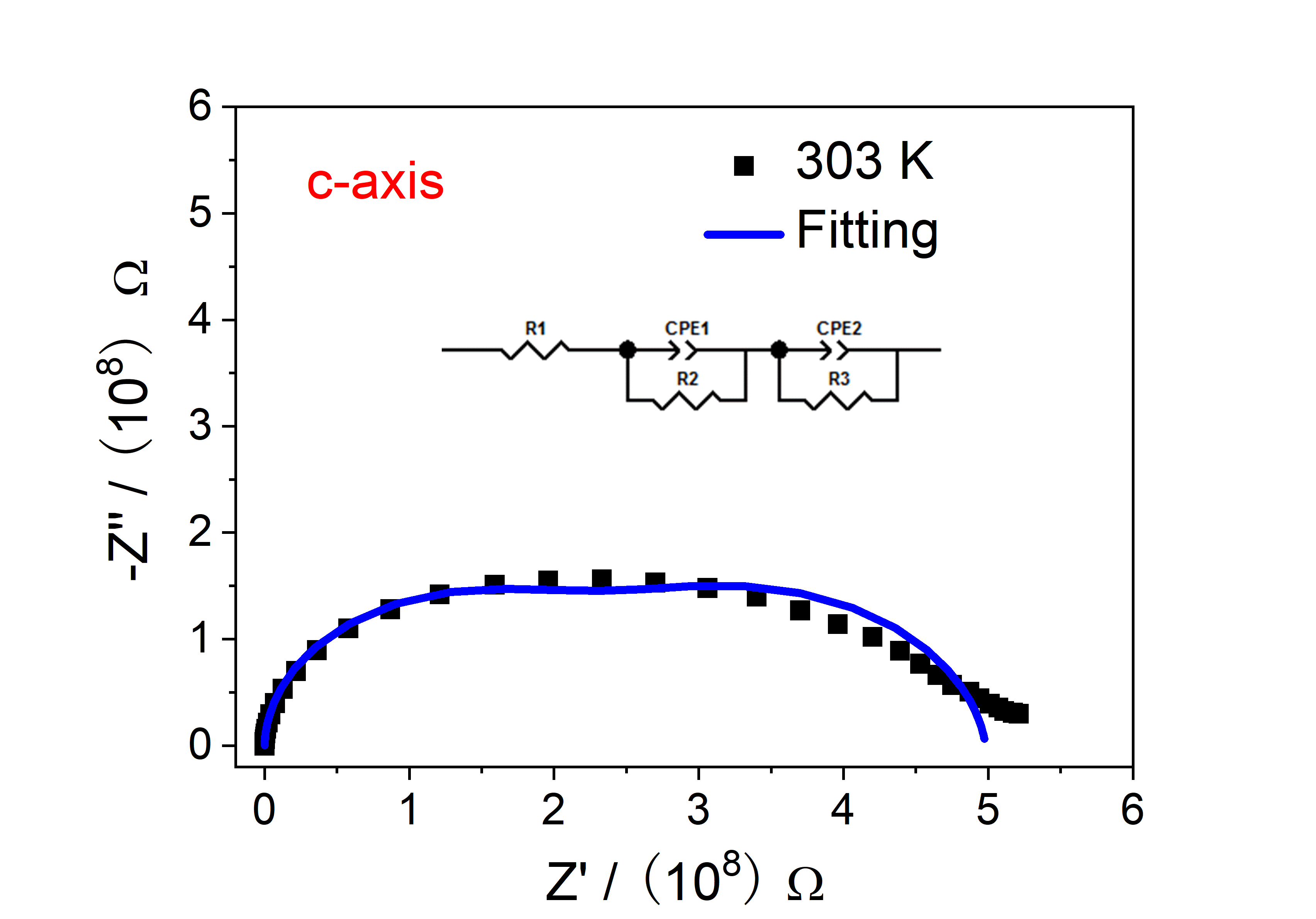

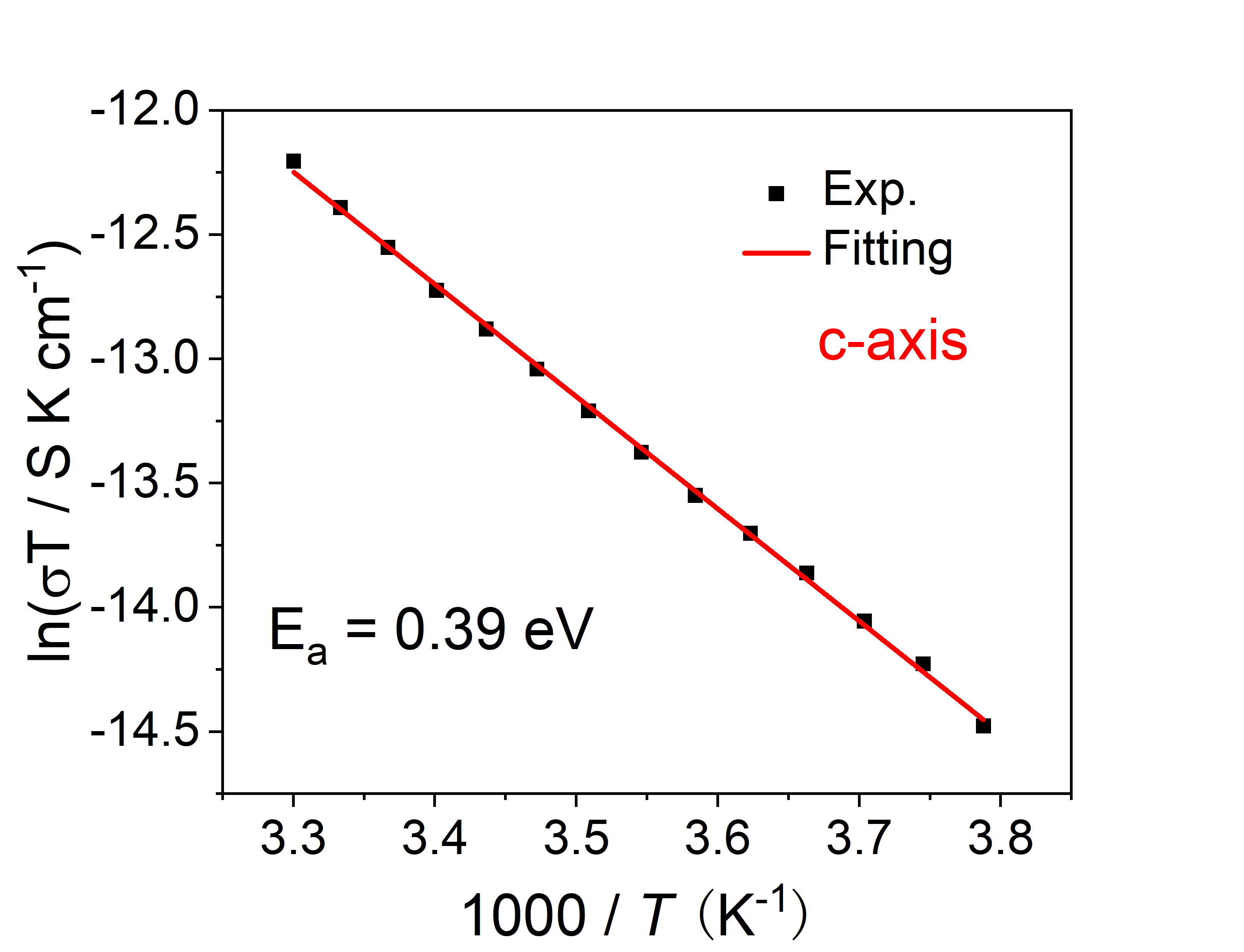


**Figure S11**: Plot of ln(σT) vs. 1000/T and the fits obtained using Arrhenius equation in the range of 273−303 K for single crystal of **1** performed along a- and c-axes, respectively.

(b)

(a)


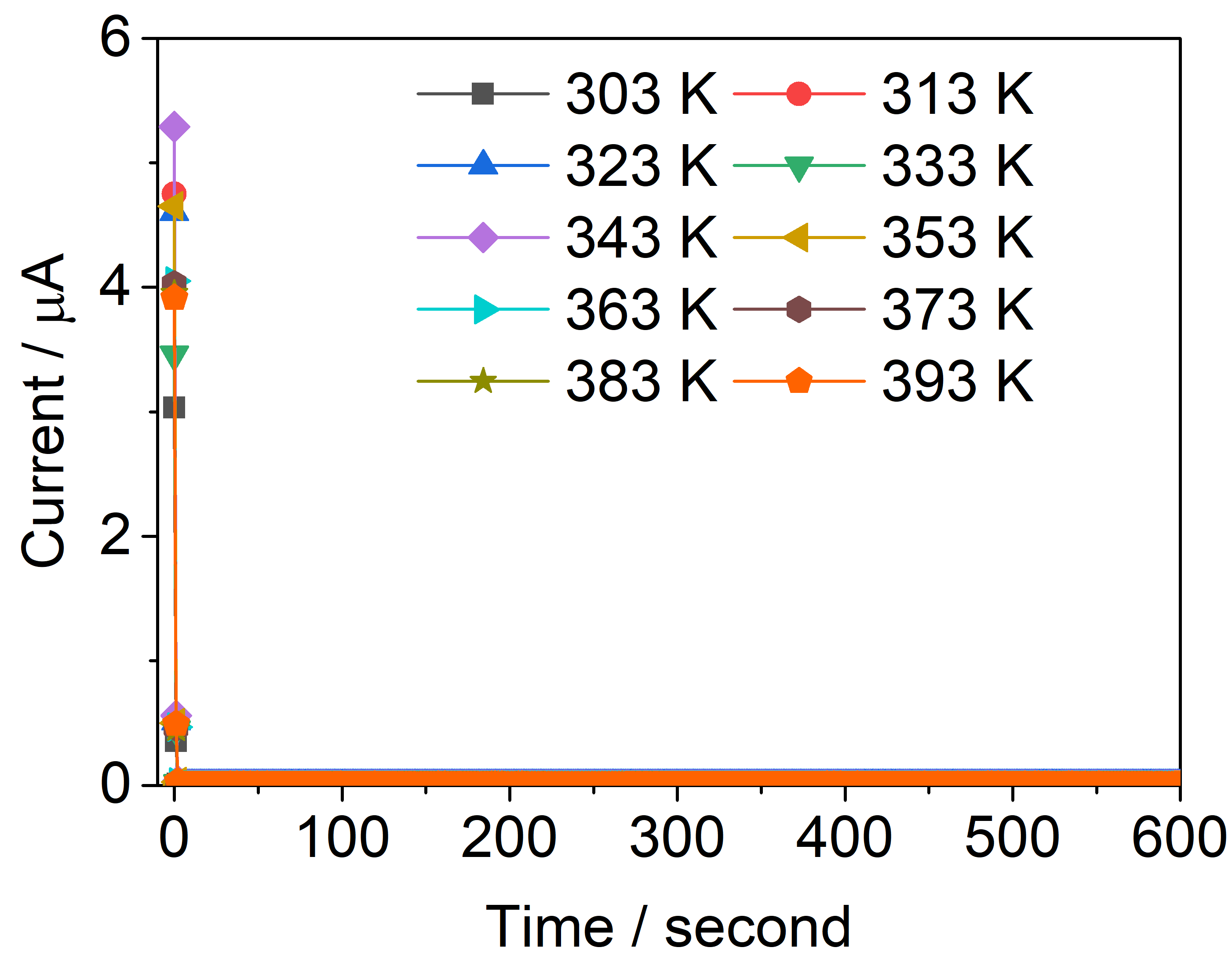

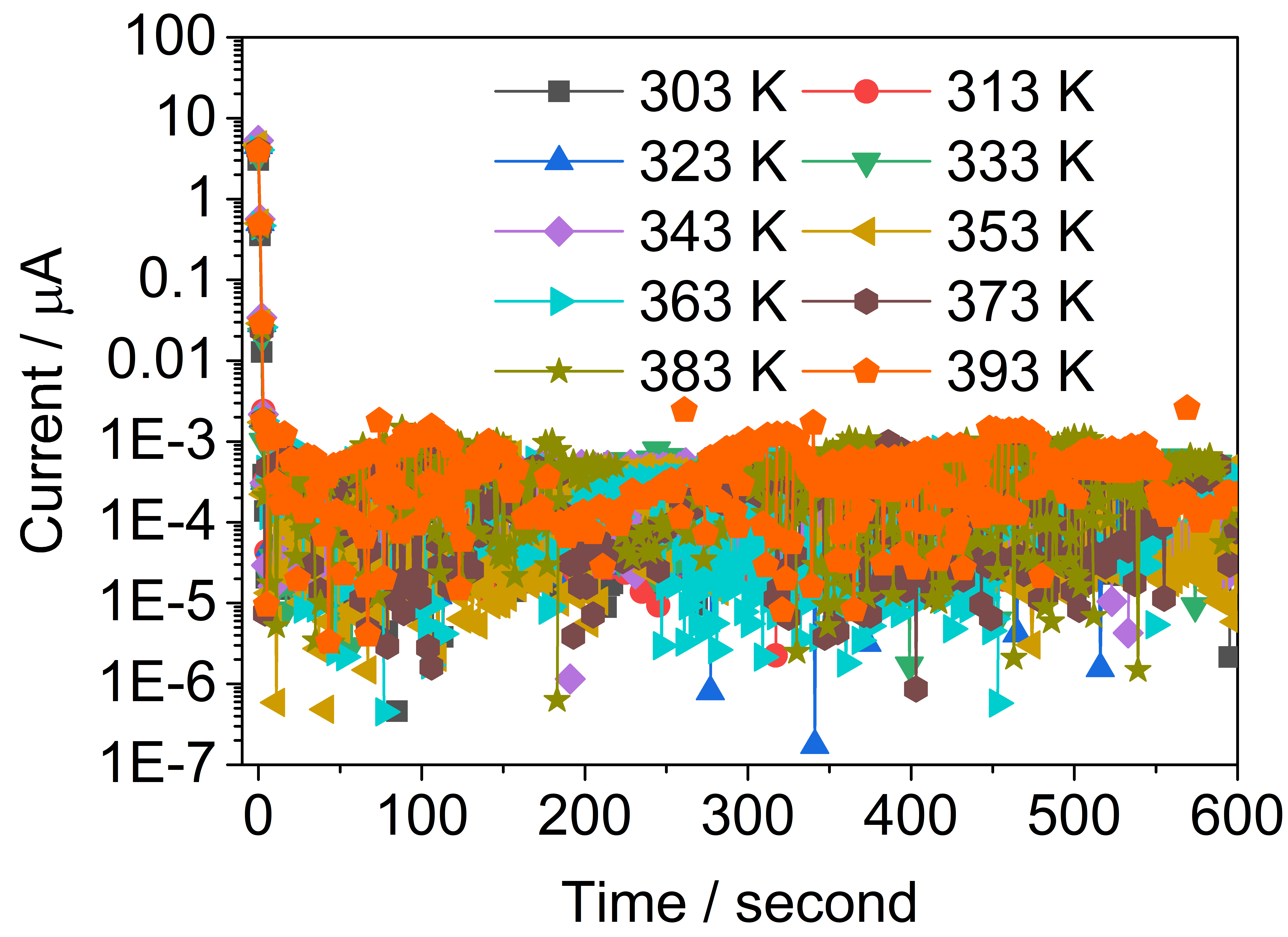


(c)

(d)


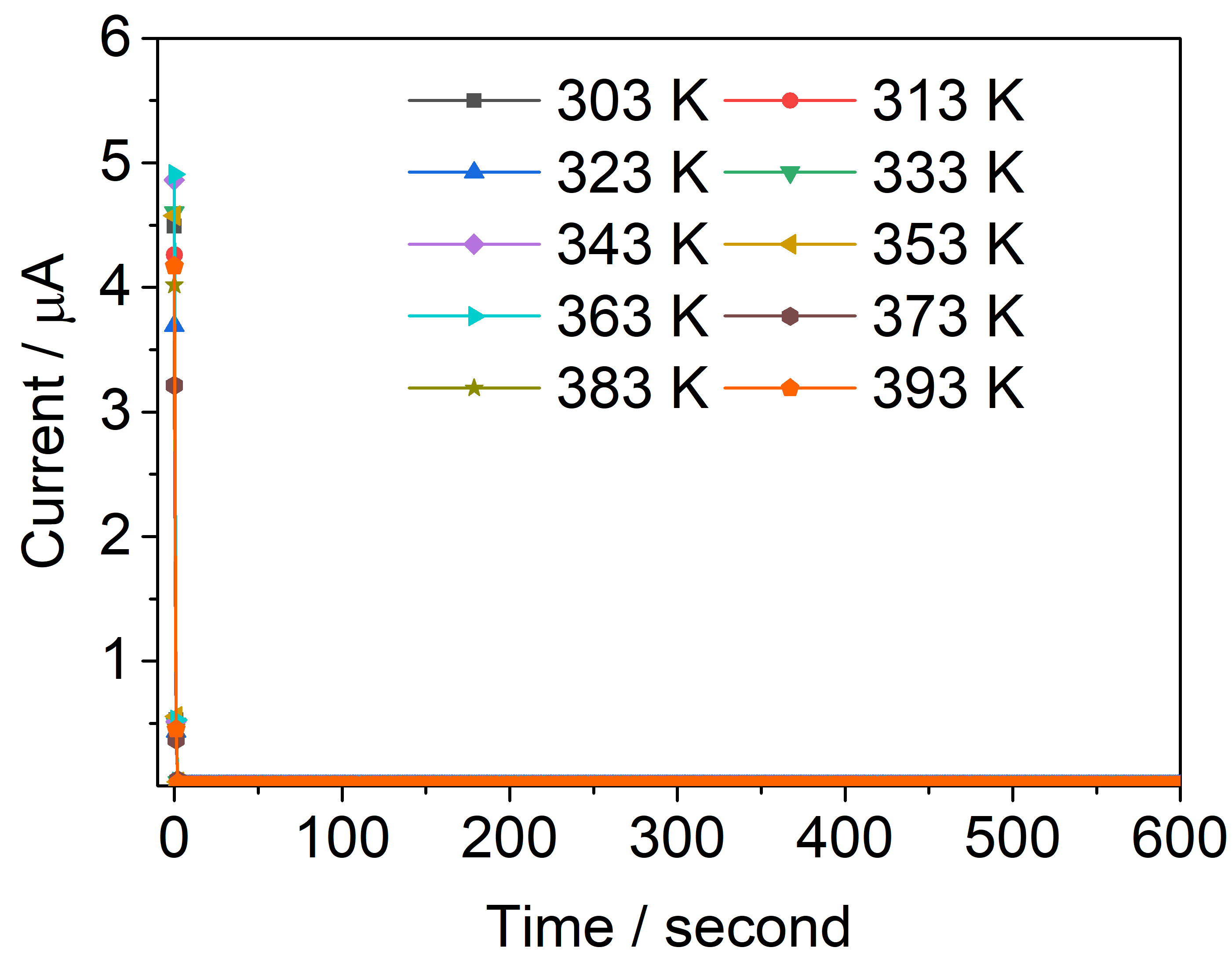

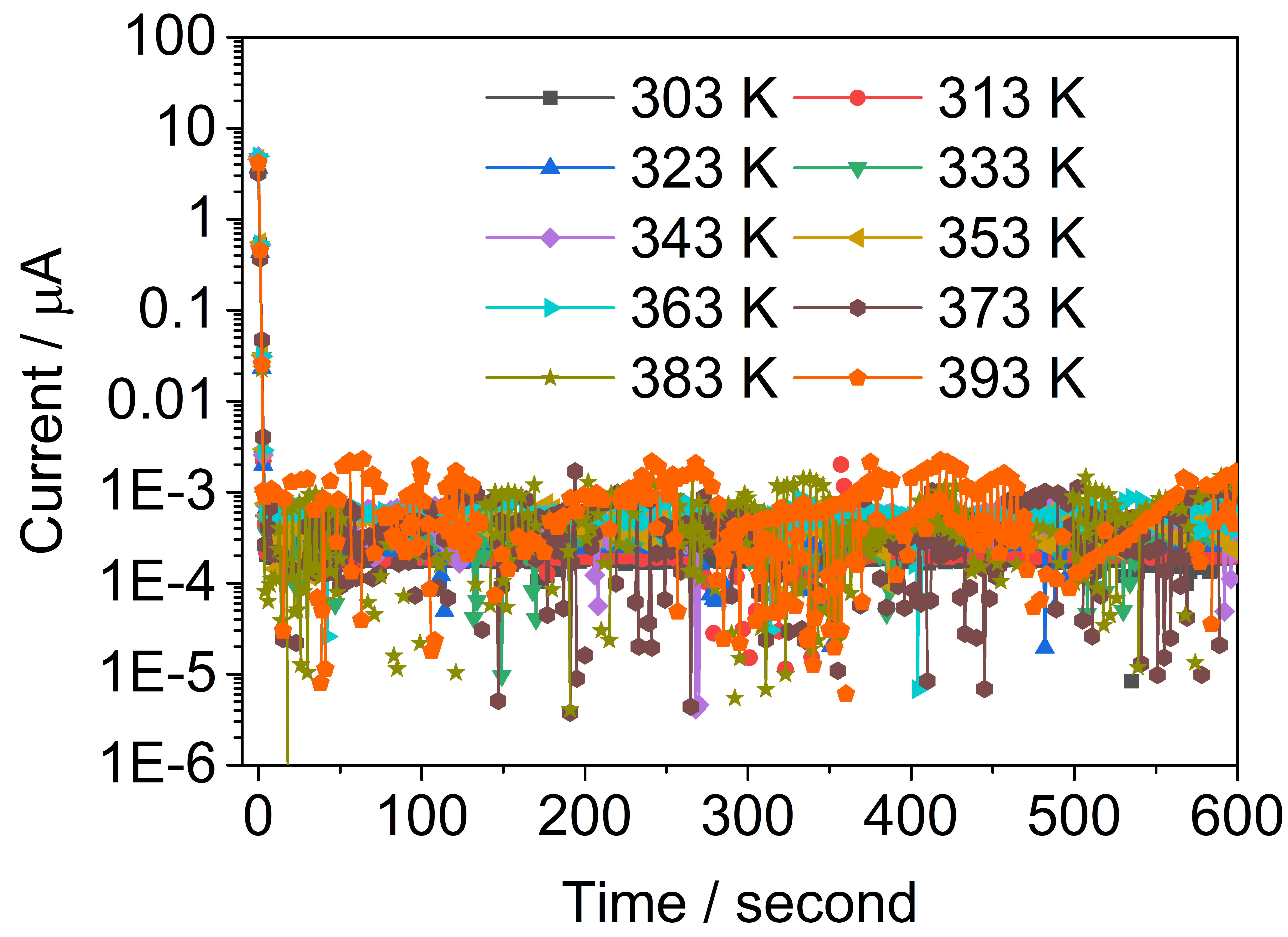


**Figure S12**: Current variation curve against time at a direct current voltage of 50 mV in the temperature range of 303−393 K for two single crystals, and the measurements along (a, b) b-axis, (c, d) c-axis of crystals, respectively.

(d)

(a)


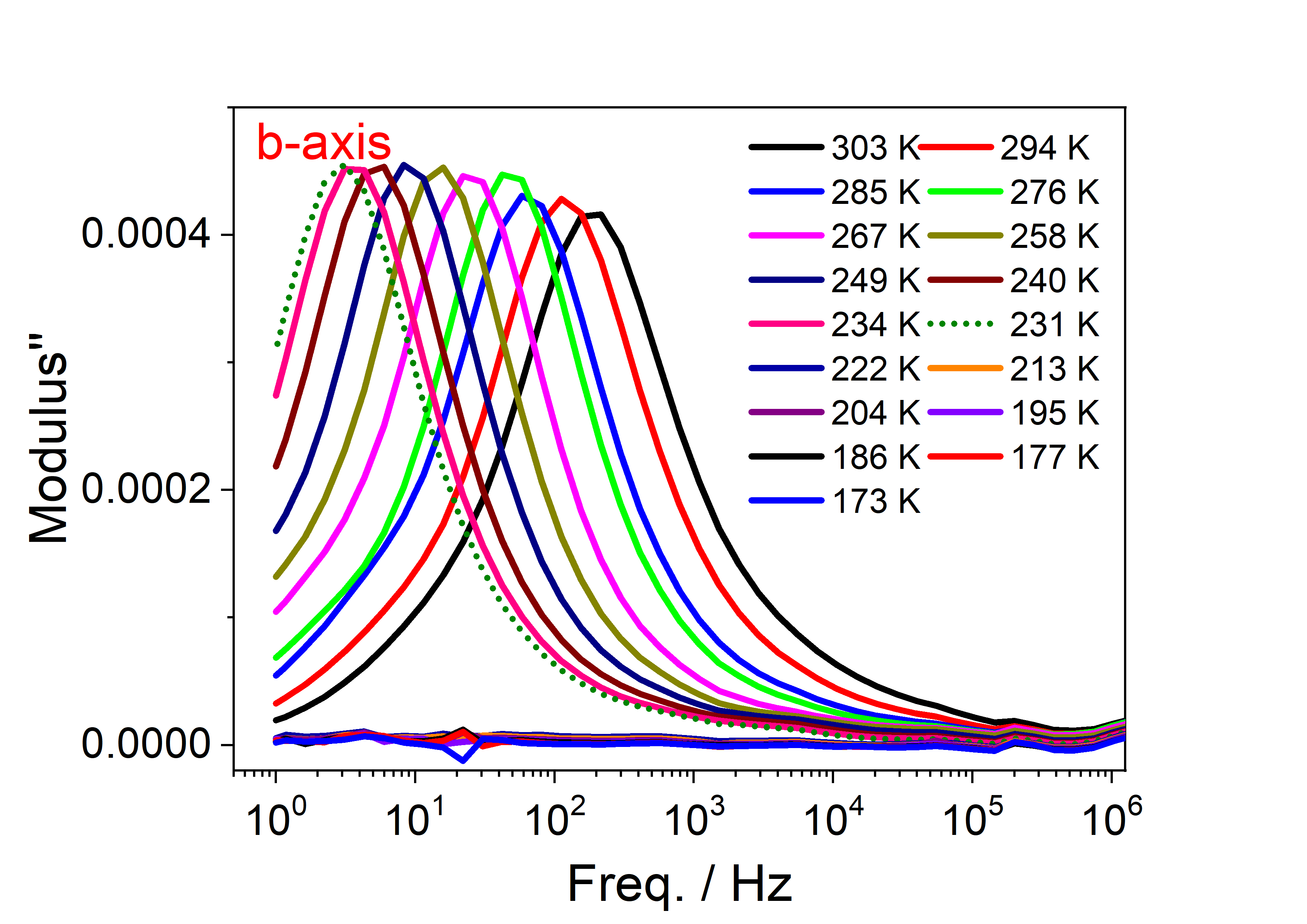

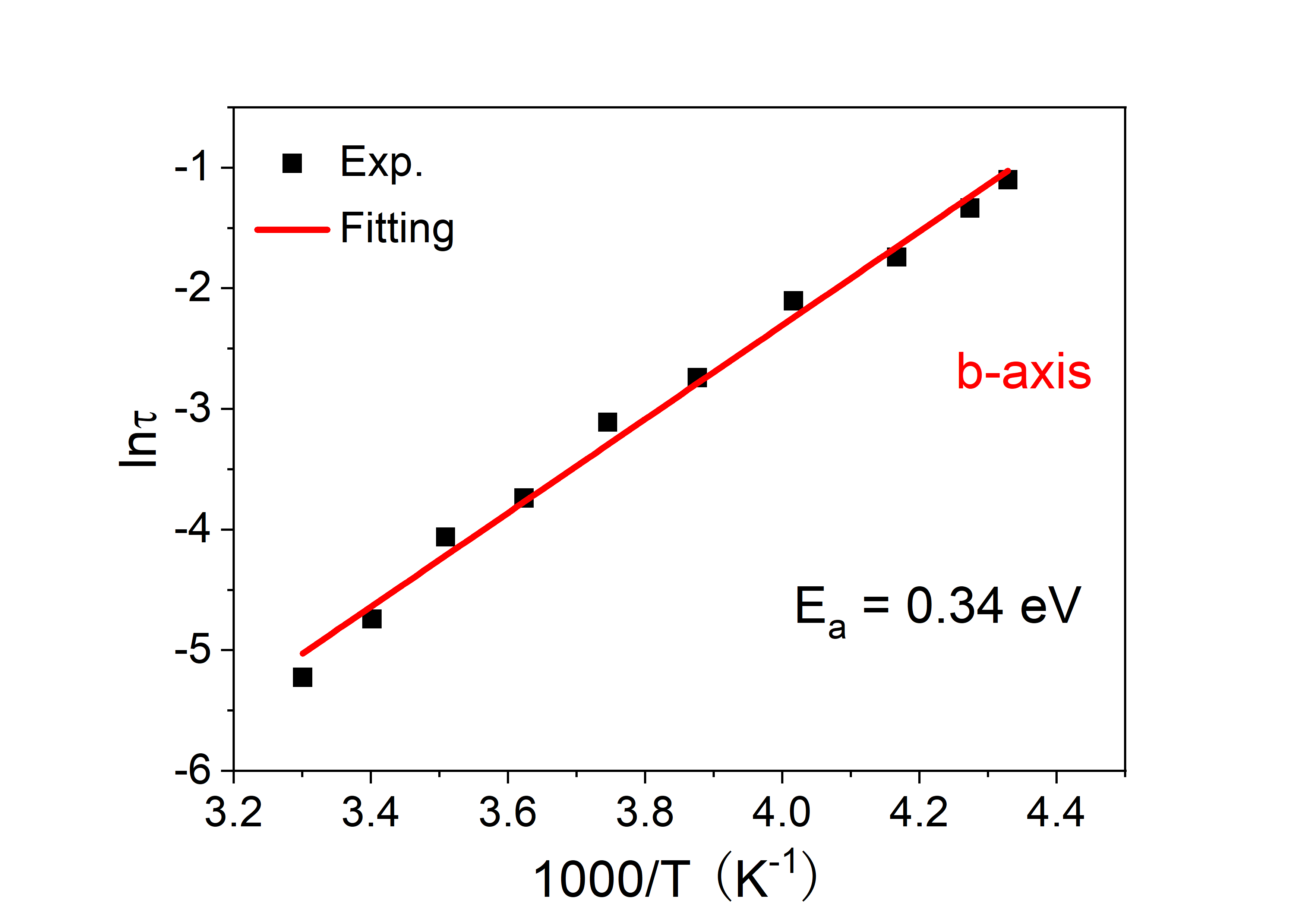


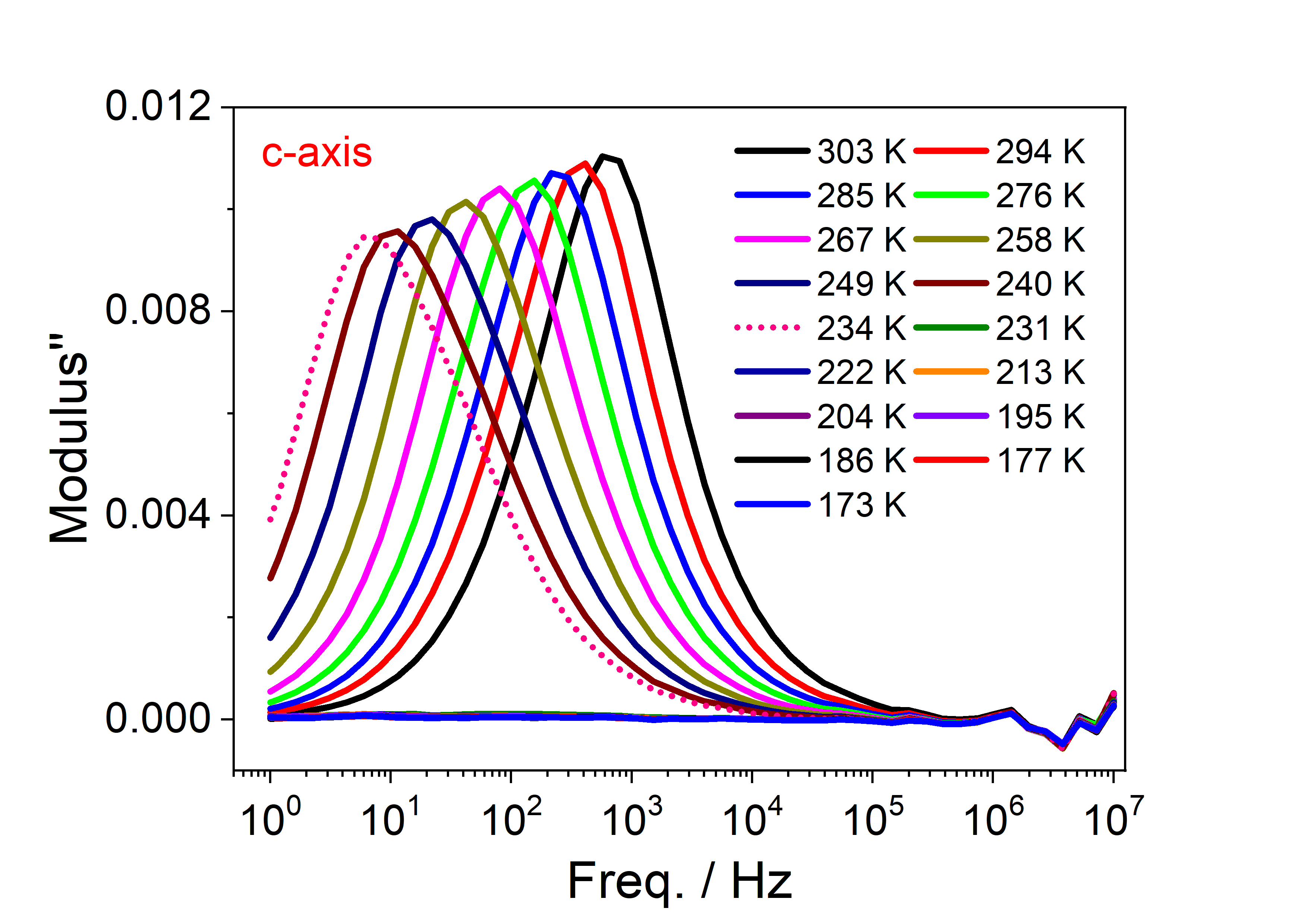

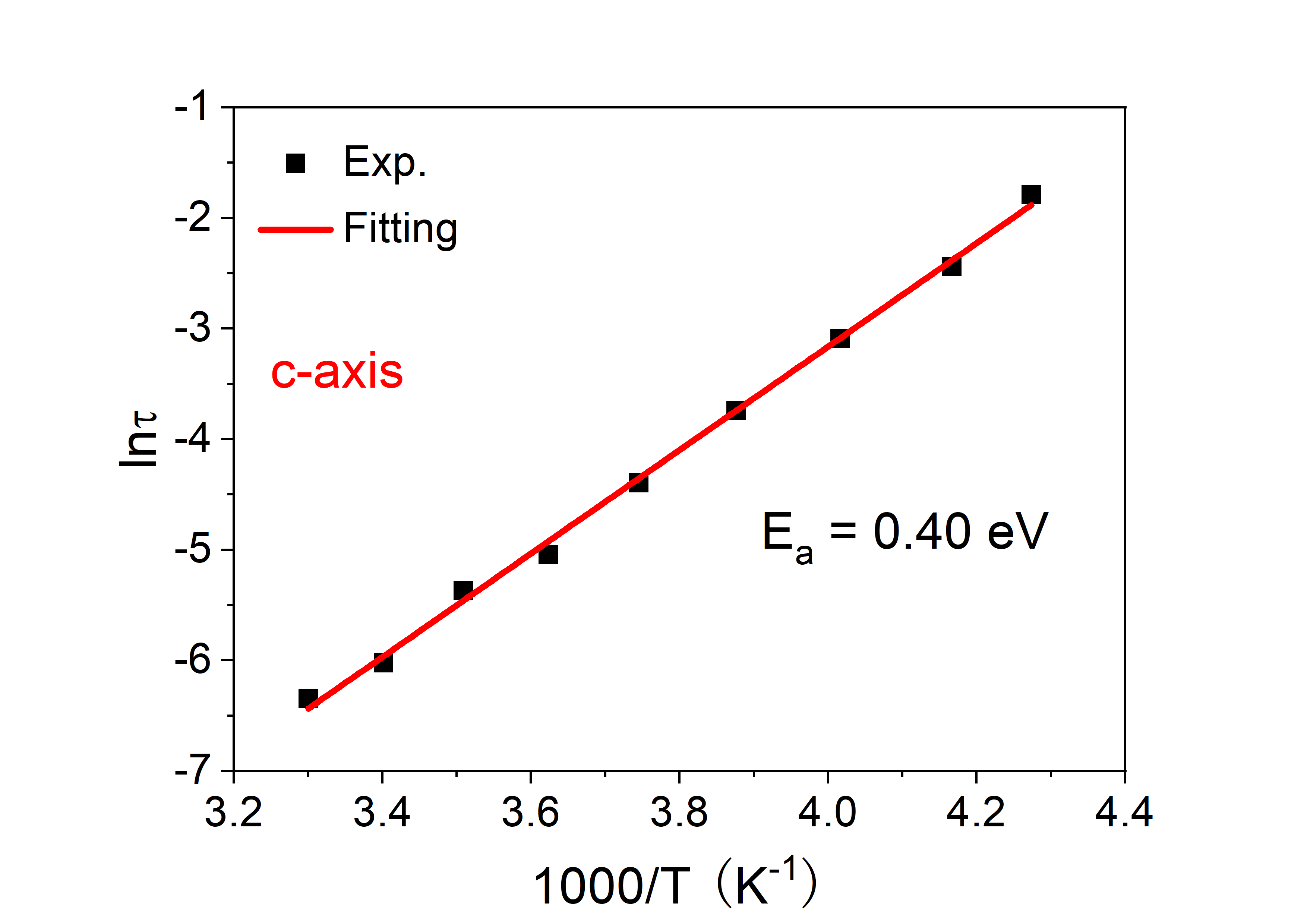

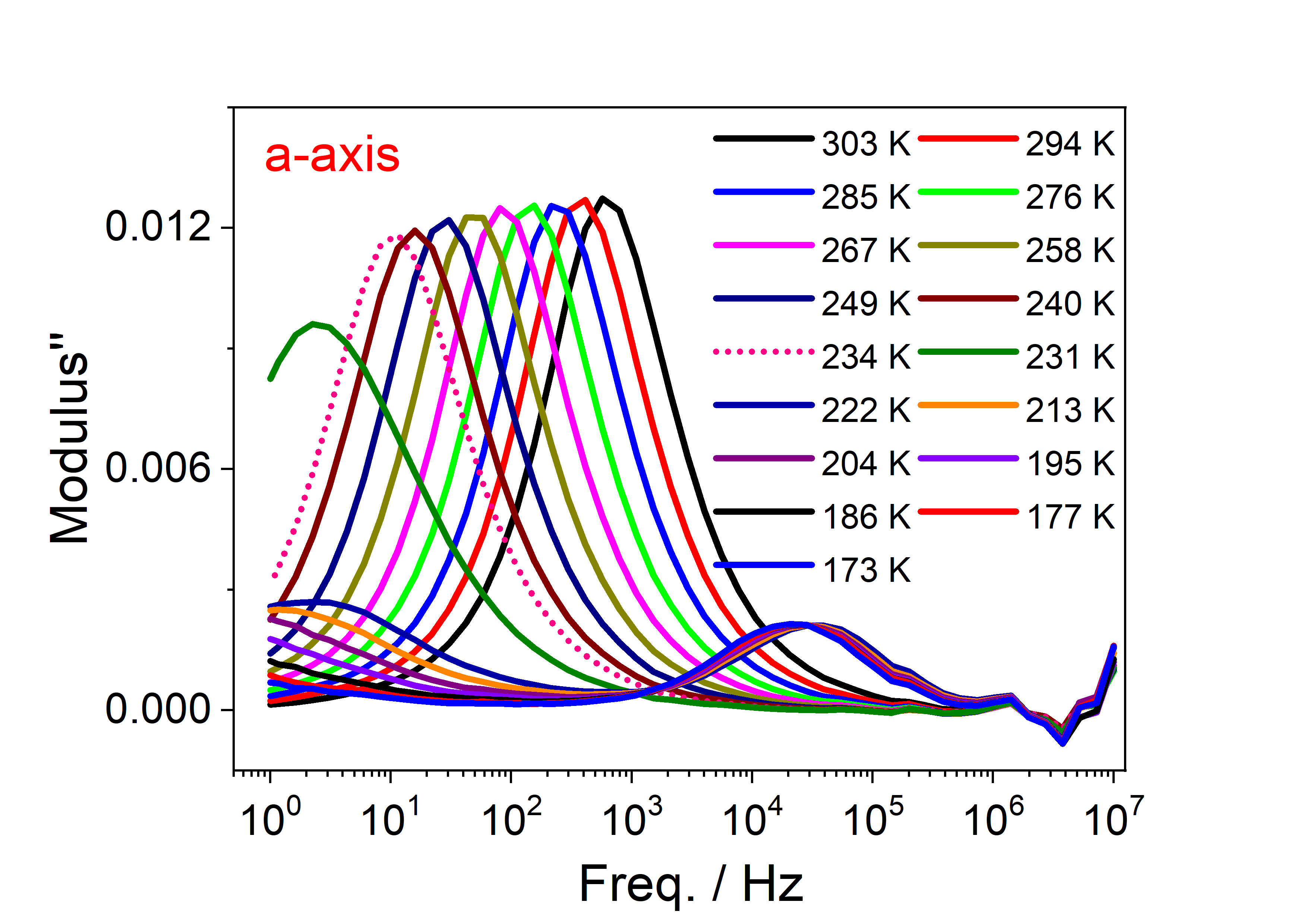

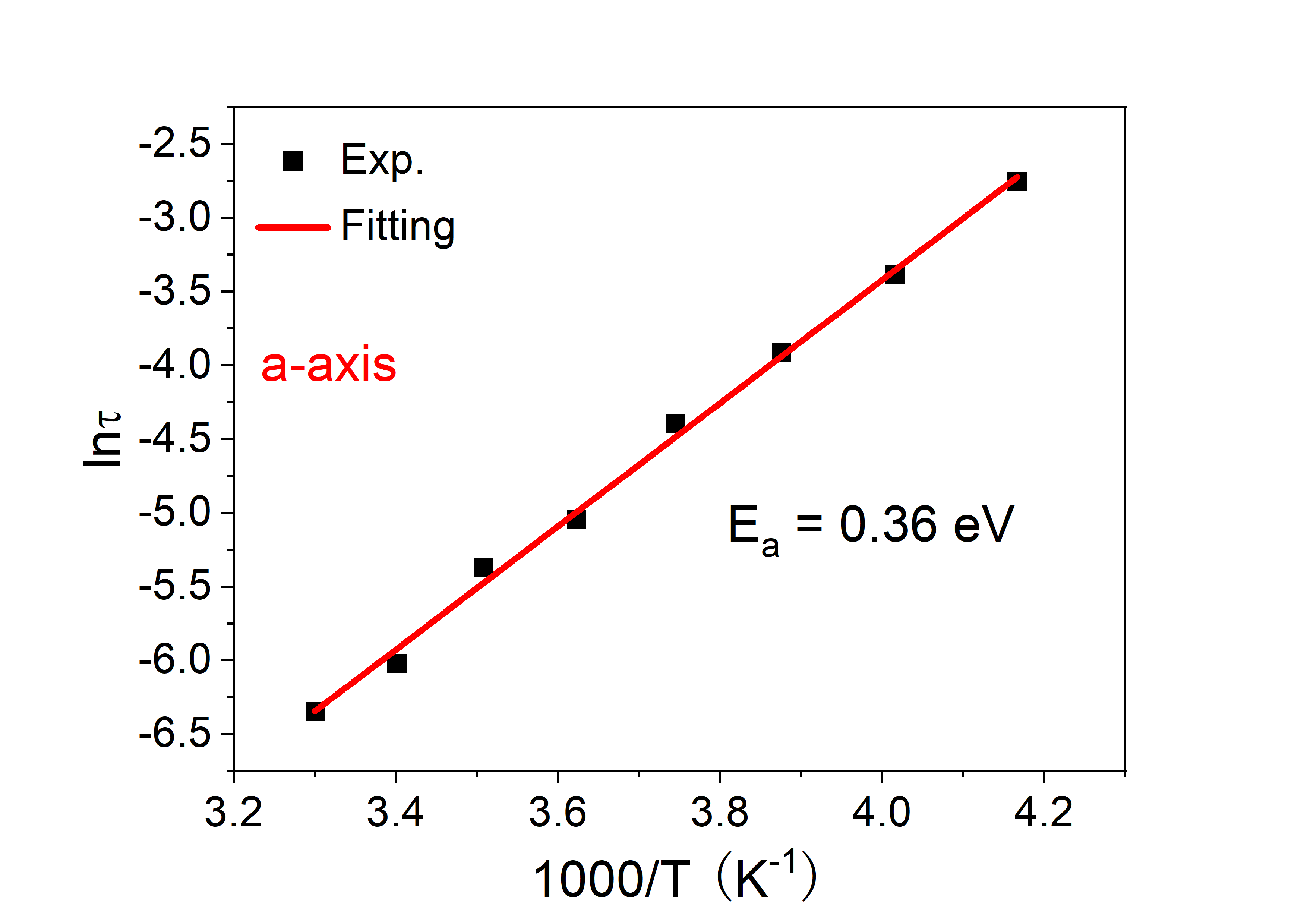


(f)

(c)

(e)

(b)

**Figure S13**: (a−c) Imaginary part of the electric modulus vs frequency at selected temperatures and (d−f) plots of lnτ vs 1000/T of **1** along b-axis, a-axis and c-axis for the relaxations in the different frequency regions.


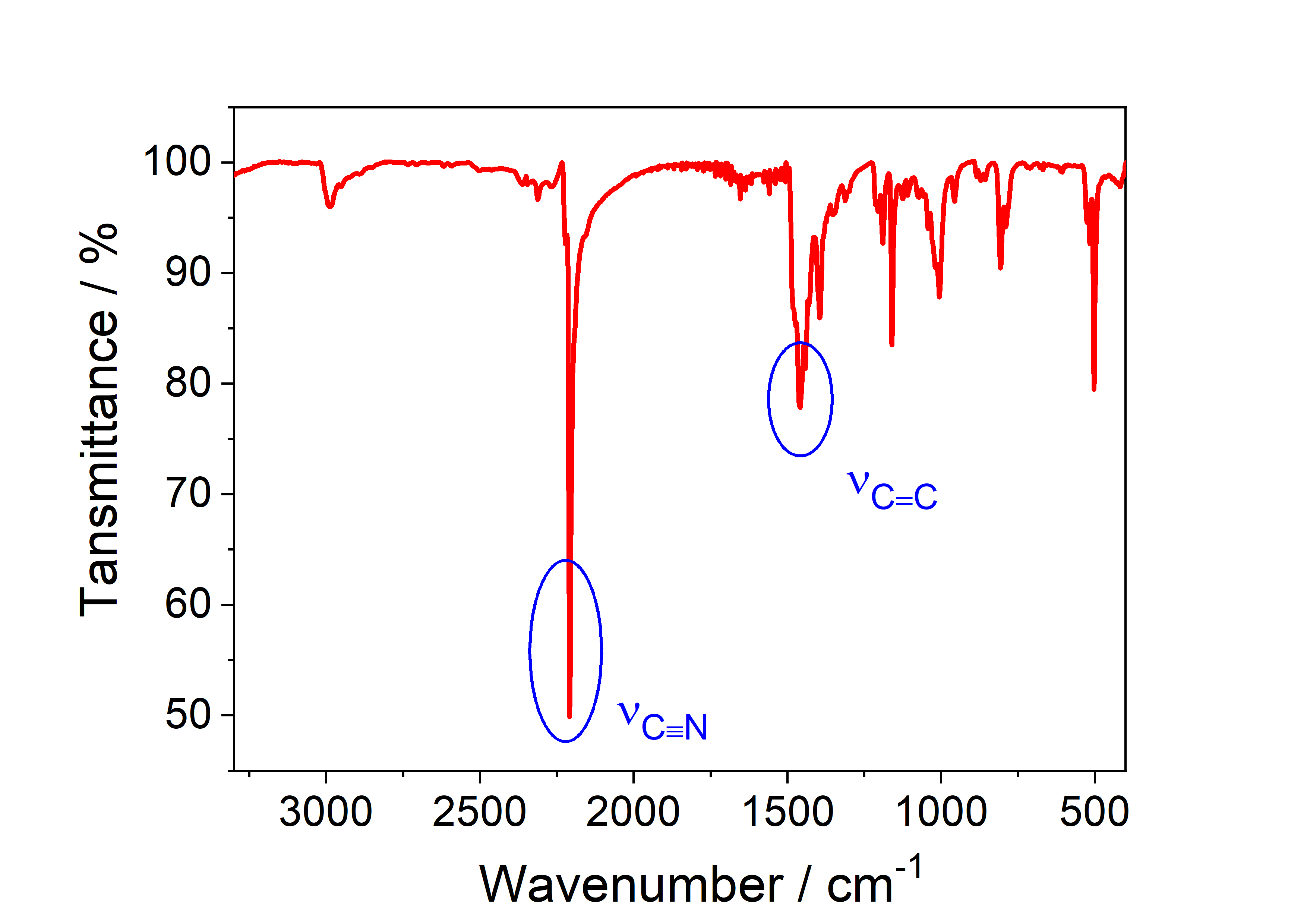


**Figure S14**: FT-IR spectrum of **1** (the main characteristic vibration bands ν_C≡N_ of the mnt^2-^ ligand (2208 cm^-1^) and ν_C=C_ of the mnt^2-^ ligand (1460 cm^-1^) are indicated).


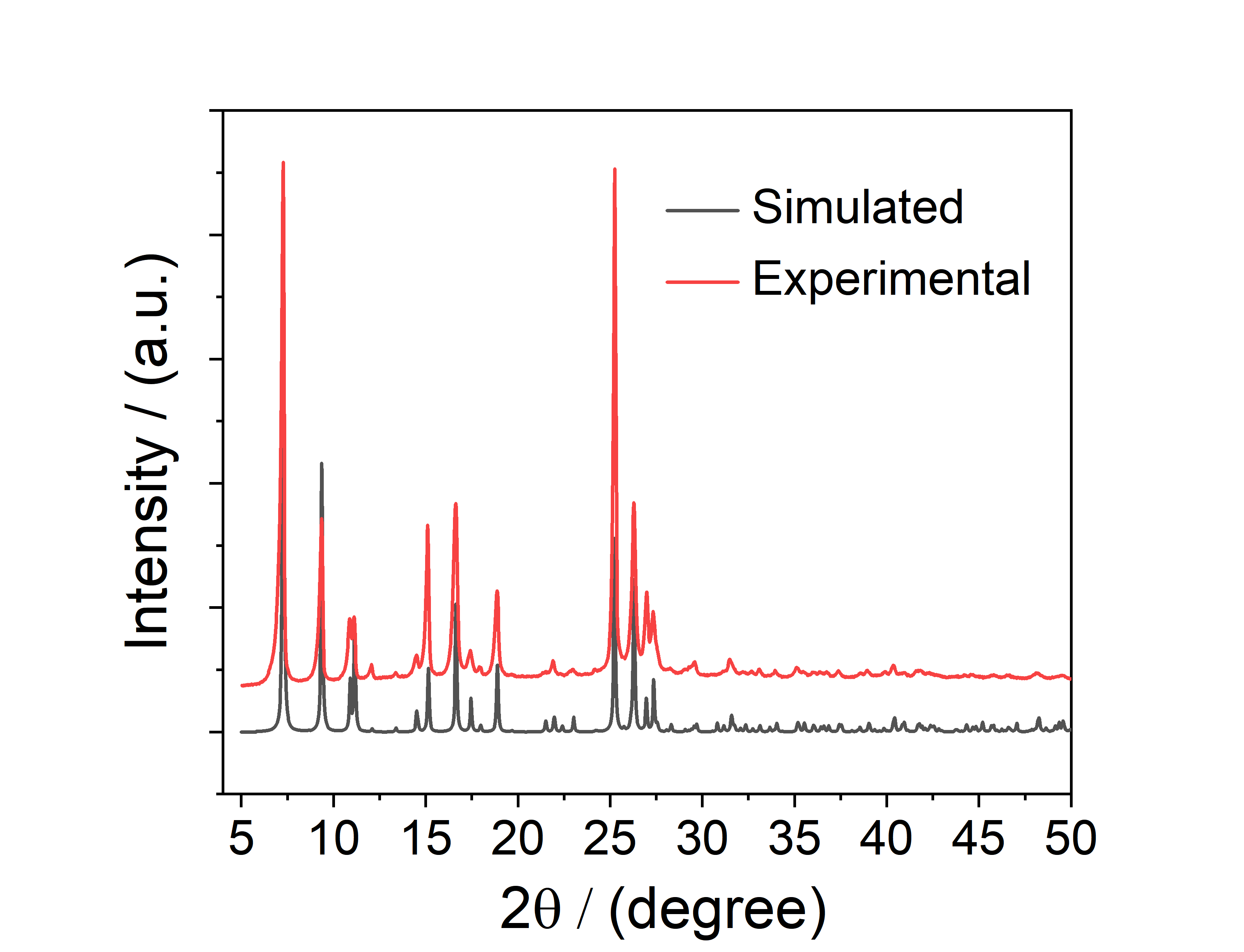


**Figure S15**: Experimental and simulated powder X-ray diffraction patterns of **1** at room temperature, indicating that the polycrystalline sample of has high phase purity.





(a)

(a)

(b)


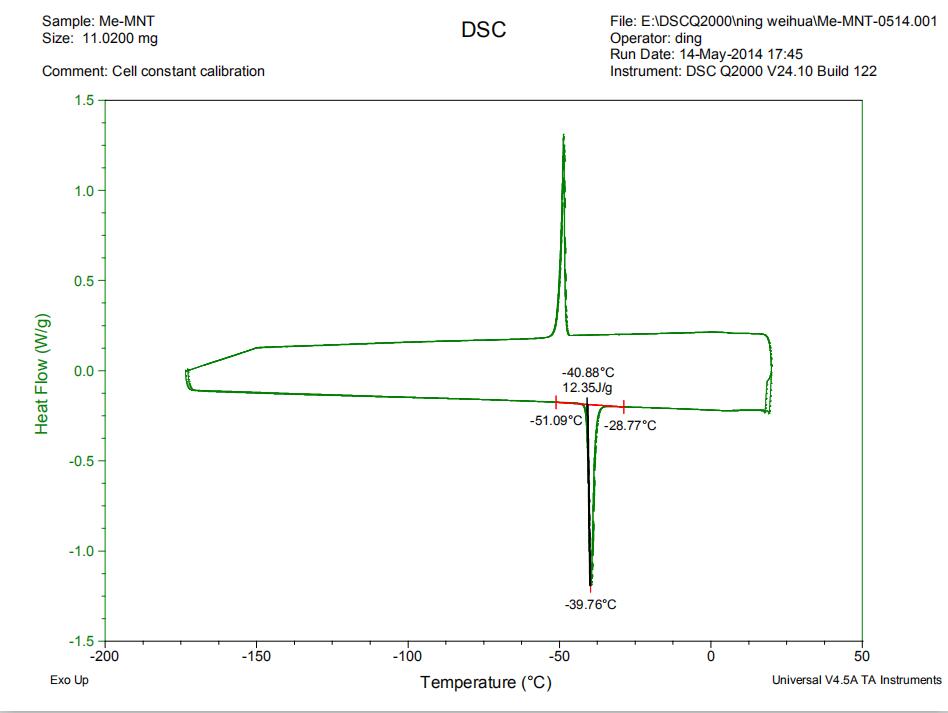


(c)


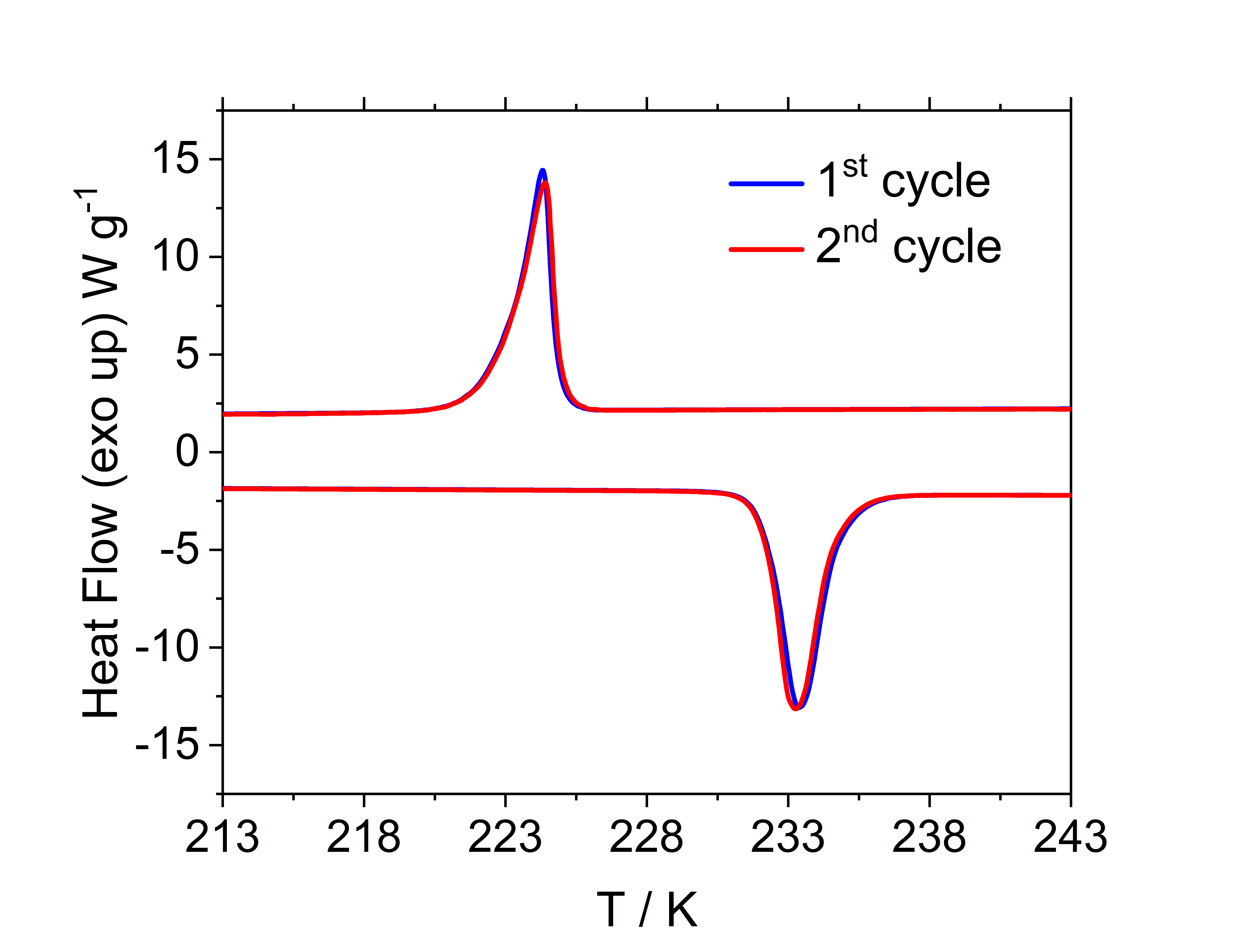


**Figure S16**: (a) TG Plots of **1** in 20−800 ºC (b, c) DSC plot of **1** between −174 and 30 ºC.

**References**

1. *Software packages SMART and SAINT*, Siemens Analytical X-ray Instrument Inc., Madison, WI, 1996.
2. G. M. Sheldrick, SHELXL-2018, *Program for the Reﬁnement of Crystal Structures*, University of Gottingen: Gottingen, Germany, **2018**.
3. K. Brandenburg and H. Putz, *Diamond*-‘Crystal and Molecular Structure Visualization’. Crystal Impact GbR, Bonn, Germany, **2006**.
